# Mediator of tolerance to abiotic stress ERF6 regulates susceptibility of Arabidopsis to *Meloidogyne incognita*

**DOI:** 10.1111/mpp.12745

**Published:** 2018-10-24

**Authors:** Sonja Warmerdam, Mark G. Sterken, Casper Van Schaik, Marian E. P. Oortwijn, Jose L. Lozano‐Torres, Jaap Bakker, Aska Goverse, Geert Smant

**Affiliations:** ^1^ Laboratory of Nematology Wageningen University Droevendaalsesteeg 1 6708 PB Wageningen the Netherlands; ^2^ Plant Breeding Wageningen University Droevendaalsesteeg 1 6708 PB Wageningen the Netherlands

**Keywords:** abiotic stress, *Arabidopsis thaliana*, ERF6, genome‐wide association mapping, *Meloidogyne incognita*, root‐knot nematodes, transcription factor

## Abstract

Root‐knot nematodes transform vascular host cells into permanent feeding structures to selectively withdraw their nutrients from host plants during the course of several weeks. The susceptibility of host plants to root‐knot nematode infections is thought to be a complex trait involving many genetic loci. However, genome‐wide association (GWA) analysis has so far revealed only four quantitative trait loci (QTLs) linked to the reproductive success of the root‐knot nematode *Meloidogyne incognita *in *Arabidopsis thaliana*, which suggests that the genetic architecture underlying host susceptibility could be much simpler than previously thought. Here, we report that, by using a relaxed stringency approach in a GWA analysis, we could identify 15 additional loci linked to quantitative variation in the reproductive success of *M. incognita* in Arabidopsis. To test the robustness of our analysis, we functionally characterized six genes located in a QTL with the lowest acceptable statistical support and smallest effect size. This led us to identify *ETHYLENE RESPONSE FACTOR 6* (*ERF6*) as a novel susceptibility gene for *M. incognita* in Arabidopsis. ERF6 functions as a transcriptional activator and suppressor of genes in response to various abiotic stresses independent of ethylene signalling. However, whole‐transcriptome analysis of nematode‐infected roots of the Arabidopsis *erf6‐1* knockout mutant line showed that allelic variation at this locus may regulate the conversion of aminocyclopropane‐1‐carboxylate (ACC) into ethylene by altering the expression of 1‐aminocyclopropane‐1‐carboxylate oxidase 3 (ACO3). Our data further suggest that tolerance to abiotic stress mediated by ERF6 forms a novel layer of control in the susceptibility of Arabidopsis to *M. incognita*.

## INTRODUCTION

Below‐ground attacks of crops by plant‐parasitic nematodes are a major constraint in global food production (Fuller et al., 2008). Outbreaks of plant‐parasitic nematodes can lead to substantial annual economic losses, amounting to $157 billion per year (Abad et al., 2008). The tropical root‐knot nematode *Meloidogyne incognita* is ranked as one of the most rapidly spreading biological threats of agricultural productivity, occurring in more than 143 countries worldwide (Bebber et al., 2014). The extensive dispersal of *M. incognita* can be attributed to its wide host range, encompassing more than 1000 plant species from 200 different genera (Perry et al., 2009; Trudgill, 1997). For decades, infestations of root‐knot nematodes in crops have been controlled by the application of chemical pesticides. However, because of regulatory bans on these compounds, the control of root‐knot nematodes has become more reliant on major resistance genes in recent years (Wesemael et al., 2011; Williamson and Kumar, 2006). Unfortunately, the panel of major genes currently available for the breeding of novel resistances to root‐knot nematodes into crops is extremely small. As a consequence of strong selection pressure by only a few widely used major resistance genes, the emergence of resistance‐breaking populations of *M. incognita* has recently turned into a major concern for growers across different continents (Davies and Elling, 2015; Kaloshian et al., 1996; Semblat et al., 2000).

Root‐knot nematodes need to establish a permanent feeding site inside a host plant to develop into the reproductive stage and to produce offspring. To this end, freshly hatched second‐stage juveniles (J2s) of *M. incognita* are attracted to the root tip of a nearby host plant. They invade the root directly above the elongation zone in the root tip. Next, invasive J2s migrate intercellularly through the cortex towards the root meristem, where they enter the vascular cylinder from below. Inside the vascular cylinder, the J2s establish a permanent feeding structure consisting of several giant cells. Giant cells are vascular cells transformed into large transfer cell‐like units. Feeding on giant cells enables J2s to moult three times into the adult female stage, whilst remaining attached to the permanent feeding structure. After a couple of weeks of feeding, adult female root‐knot nematodes produce offspring as an aggregate of eggs held together by a gelatinous matrix (Caillaud et al., 2008; Gheysen and Mitchum, 2011; Kyndt et al., 2013).

Whole‐transcriptome analyses of giant cell‐enriched tissue of roots infected with root‐knot nematodes show that feeding site formation is accompanied by the differential regulation of more than 1000 genes (Barcala et al., 2010; Fuller et al., 2007; Jammes et al., 2005). The large number of genes regulated in association with feeding site formation suggests that the reproductive success of *M. incognita* might be a highly polygenic trait in plants. However, it should be noted that many genes may be regulated in response to the massive cellular changes induced by feeding nematodes, but may not necessarily be required for the susceptibility of plants to nematode infections. Nonetheless, allelic variation in genes that do enable initiation, expansion and maintenance of giant cells may translate into quantitative variation in susceptibility to nematode infections. If this holds true, gain‐ and loss‐of‐function alleles of these so‐called susceptibility genes may be used in the future to make crops more resilient to nematode infections (de Almeida Engler et al., 2005; van Schie and Takken, 2014).

Previously, we have used a genome‐wide association (GWA) mapping approach to assess whether quantitative variation in susceptibility to *M. incognita* can be linked to allelic variation in specific loci (quantitative trait loci, QTLs) in Arabidopsis (Warmerdam et al., 2018). In that study, we found four QTLs significantly associated with the number of egg masses of *M. incognita* per plant at 6 weeks post‐inoculation. We noted that, by using a threshold for significant associations of –log_10_(*P*) = 5, the accumulated effect sizes of the alleles in these four loci could account for only 50% of the heritable variation in susceptibility of Arabidopsis to *M. incognita*. We also noted that reducing this threshold to the level of −log_10_(*P*) = 4 would have explained all of the heritable variation in susceptibility of Arabidopsis to *M. incognita*. However, relaxing the stringency of our GWA approach would also have increased the risk of pursuing false positives. Here, we describe how we challenged this higher risk of false discovery under relaxed criteria by functionally characterizing six genes located at QTL13, which, in terms of statistical support and effect size, had a relatively high chance of being a false positive. This led us to identify *ETHYLENE RESPONSE FACTOR 6* (*ERF6*) as a regulator of susceptibility to *M. incognita* in Arabidopsis. The transcription factor ERF6 has not been associated previously with susceptibility to *M. incognita*, but its role as an activator and repressor of abiotic stress response genes may shed new light onto the role of tolerance to abiotic stress in feeding site formation.

## RESULTS

Stepwise lowering of the threshold for significant associations in our original analysis from −log_10_(*P*) = 5 to −log_10_(*P*) = 4 revealed 28 additional single nucleotide polymorphisms (SNPs), resulting in a total number of 36 SNPs significantly associated with the number of egg masses of *M. incognita* per plant (Fig. S1; Table S1, see Supporting Information). Of this set of 36 SNPs, 12 showed strong linkage (*R* ^2^ > 0.8): four SNPs around position Chr1:28.18Mb, six SNPs around position Chr5:6.26Mb and two SNPs around position Chr5:22.68Mb (Fig. S2, see Supporting Information). By using the predicted average decline in linkage disequilibrium (LD) in Arabidopsis within 10 kb (Kim et al., 2007), we aggregated the remaining SNP markers into 19 QTLs (Table 1), two of which have already been described in more detail in Warmerdam et al. (2018). It should be noted that multiple SNPs aggregated into the same QTL when their LD regions overlapped, resulting in four QTLs spanning more than 20 kb. Altogether, by accepting −log_10_(*P*) = 4 as a threshold for significance, we could map all of the allelic variation (in 19 QTLs) underlying the heritable phenotypic variation in the reproductive rate of *M. incognita* in our population of Arabidopsis lines.

**Table 1:** Details of 36 single nucleotide polymorphisms (SNPs) significantly associated with the reproductive success of *Meloidogyne incognita* aggregated into 19 quantitative trait loci (QTLs).

QTL*	Chromosome	Position (bp)	Alleles	SNP ratio†	−log_10_(*P*)‡	Effect size	SNP located in gene	Genes in 20‐kb LD
5	1	10540036	A : G	34 : 315	4	5.95	At1G30050	At1G30030, At1G30040, At1G30050, At1G30060, At1G30070, At1G30080
6	1	17480468	C : T	149 : 200	4.5	3.40	At1G47570	At1G47560, At1G47565, At1G47570, At1G47578, At1G47580, At1G47590, At1G47595
1	1	28186622	G : T	268 : 81	4	4.86	At1G75080	At1G75030, At1G75040, At1G75050, At1G75060, At1G75070 At1G75080, At1G75090, At1G75100, At1G75110, At1G75120, At1G75130
		28187392	C : G	278 : 71	5.1	5.62	At1G75080	
		28187959	C : T	247 : 102	4.7	4.96	At1G75080/At1G17090	
		28187978	A : T	247 : 102	4.7	4.96	At1G75080/At1G17090	
		28188103	C : T	104 : 245	4.4	4.73	At1G75090	
		28188151	C : G	102 : 247	5.1	5.10	At1G75090	
7	2	14465496	A : C	107 : 242	4	4.39	NA	At2G34230, At2G34238, At2G34240, At2G34250, At2G34260, At2G34270, At4G34280, At2G34290, At2G34300
8	2	14848612	G : T	150 : 199	4	4.28	At2G35250	At2G35215, At2G35220, At2G35230, At2G35240, At2G35250. At2G35260, At2G35270
9	2	18338888	A : G	211 : 138	4.6	4.56	At2G44440	At2G44400, At2G44410, At2G44420, At2G44430, At2G44440, At2G44450, At2G44460
10	3	287272	C : T	131 : 218	4.4	4.19	At3G01800	At3G01770, At3G01780, At3G01790, At3G01800, At3G01810, At3G01820, At3G01830. At3G01840
11	3	21020421	C : G	130 : 219	4.6	3.48	At3G56750	At3G56720, At3G56740, At3G56750, At3G56760, At3G770
12	4	7211220	C : T	104 : 245	4.2	4.15	At4G12030	At4G12010, At4G12020, At4G12030, At4G12040, At4G12050
13	4	9765013	C : T	133 : 216	4	3.83	At4G17505	At4G17490, At4G17500, At4G17505, At4G17510, At4G17520, At4G17530
14	4	9843096	C : T	96 : 253	4.1	4.30	At4G17680	At4G17660, At4G17670, At4G17670, At4G17680, At4G17690, At4G17695
15	4	14391100	A : G	108 : 241	4.3	3.36	NA	At4G29160, At4G29170, At4G29180, At4G29190, At4G29200, At4G29210
2	5	6250487	A : G	139 : 210	4	4.62	At5G18740	At5G18700, At5G18710, At5G18720, At5G18730, At5G18740, At5G18748, At5G18750, At5G18755, At5G18760, At5G18770, At5G18780, At5G18790, At5G187800, At5G18810, At5G18820
		6253982	C : T	202 : 147	4.7	4.84	NA	
		6261603	A : T	109 : 240	4.5	4.88	At5G18770	
		6263591	A : T	139 : 210	6.1	5.31	At5G18780	
		6263577	A : T	139 : 210	6.1	5.31	At5G18780	
		6263644	A : T	140 : 209	5.7	5.18	At5G18780	
		6263678	C : G	139 : 210	6.1	5.31	At5G18780	
16	5	14116311	A : T	253 : 96	4	5.05	At5G35965	At5G35650, At5G35960, At5G35965, At5G35970
17	5	14139771	C : G	218 : 131	4	4.54	NA	At5G35995, At5G36001, At5G36002, At5G36005, At5G36010, At5G36015. At5G36020, At5G36030
		14152182	C : T	179 : 170	4.7	4.65	NA	
		14156930	G : T	210 : 139	4.5	4.31	At5G36015	
3	5	14913458	A : T	288 : 61	5.5	5.35	At5G37540	At5G37520, At5G37530, At5G37540, At5G37550, At5G37560
4	5	15904331	C : T	290 : 59	6	4.47	At5G39740	At5G39700, At5G39710, At5G39720, At5G39730, At5G39740, At5G39750, At5G39760
18	5	22583411	C : G	19 : 330	4.6	8.30	At5G55800	At5G55770, At5G55780, At5G55790, At5G55800, At5G55810, At5G55820, At5G55830, At5G55835, At5G55840, At5G55850, At5G55855, At5G55856, At5G55860
		22601633	A : C	17 : 332	4	7.95	At5G55840	
		22602265	G : T	17 : 332	4.3	8.07	At5G55840	
						
19	5	22668012	A : G	63 : 286	4.9	5.24	At5G55970	At5G55940, At5G55950, At5G55960, At5G55970, At5G55980, At5G55990, At5G56000, At5G56010, At5G56020, At5G56030 At5G56040
		22683923	C : G	31 : 318	4	5.72	At5G56010	
		22684613	C : T	29 : 320	4.1	5.89	At5G56020	

*QTL1 to QTL4 have been identified previously (Warmerdam et al., 2018).

†Ratio of alleles within the population of 349 Arabidopsis natural inbred lines.

‡Level of significance of the association of individual SNPs and number of egg masses of *M. incognita* per plant.

**Figure 1:**
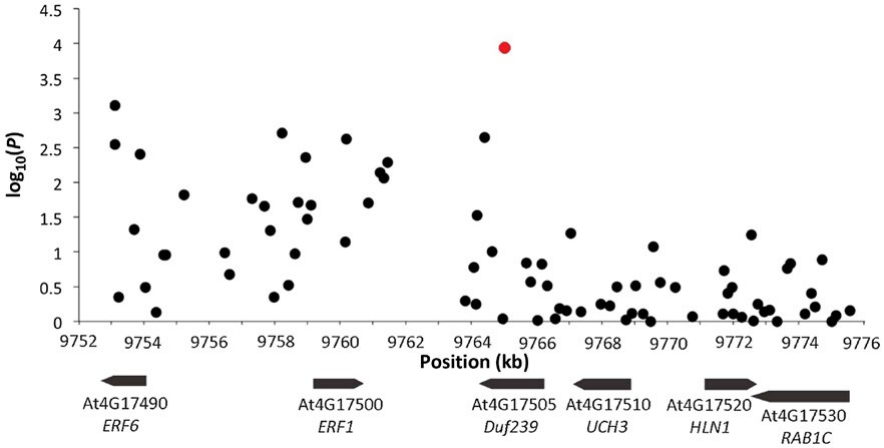
Genome region surrounding the single nucleotide polymorphism (SNP) marker Chr4.145290 (red dot) on chromosome 4 of Arabidopsis. The black dots represent the SNPs located in the 10‐kb region up‐ and downstream of Chr4.145290. The vector arrows represent the predicted genes in this region with accession numbers and abbreviations of the corresponding names. [Colour figure can be viewed at wileyonlinelibrary.com]

The cost associated with the lowering of the threshold for significance is an increase in the false discovery rate to 0.55. To challenge this high risk of false positive SNPs, we investigated QTL13 marked by only one SNP (Chr4.145290) with significance on the threshold value [−log_10_(*P*) = 4] and within the subset with the smallest effect size (3.83 egg masses). SNP marker Chr4.145290 is located at genomic position 9765013 of chromosome 4 inside the fifth predicted intron of gene model At4G17505 (Fig. 1). At4G17505 is annotated as a gene encoding a putative protein domain with unknown function (*DUF239*). With the average LD decline in Arabidopsis (±10 kb) in mind, we identified in total six predicted genes that could be linked to SNP marker Chr4.145290, and which could therefore contribute to the observed variation in the number of egg masses of *M. incognita* per plant at 6 weeks post‐inoculation.

### ERF6 regulates the susceptibility of Arabidopsis to *M. incognita*

**Figure 2:**
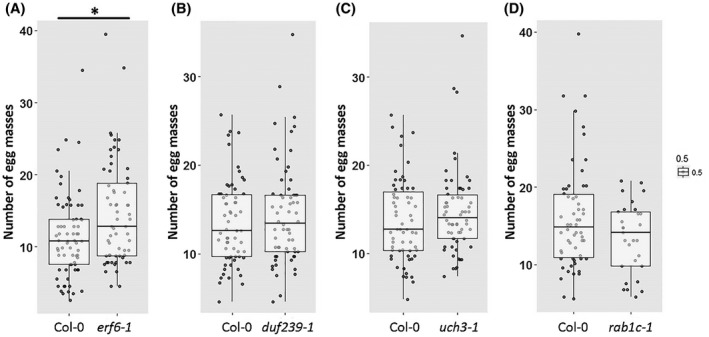
The number of egg masses of *Meloidogyne incognita* per plant on homozygous T‐DNA insertion mutants *erf6‐1* (ERF6), *duf239‐1* (DUF239), *uch3‐1* (UCH3) and *rab1c‐1* (RAB1C) of Arabidopsis at 6 weeks post‐inoculation. (A) Number of egg masses on *erf6‐1* and wild‐type Arabidopsis plants. (B) Number of egg masses on *duf239‐1* and wild‐type Arabidopsis plants. (C) Number of egg masses on *uch3‐1* and wild‐type Arabidopsis plants. (D) Number of egg masses on *rab1c‐1* and wild‐type Arabidopsis plants. Data points were obtained from three independent experiments with a total of *n* > 30. Data were statistically analysed using analysis of variance (ANOVA) and *post‐hoc* Tukey’s honestly significant difference (HSD) test (*P < 0.05).

To determine which of the genes predicted for QTL13 could play a role in the reproductive success of *M. incognita* in Arabidopsis, we investigated several homozygous T‐DNA insertion lines available for this locus. First, we tested an Arabidopsis line harbouring a T‐DNA insert in *DUF239*, which harbours SNP marker Chr4.145290 (*DUF239*), but which was not significantly altered in susceptibility to *M. incognita* compared with wild‐type Arabidopsis plants (Fig. 2B). The T‐DNA insert in this line is located at the predicted 5’‐end of the coding sequence of At4G17505, but this does not seem to affect the amplification of reverse transcribed mRNA matching the sequence further to the 3’‐end (Fig. S2B). It should be noted that the gene model At4G17505 harbouring SNP marker Chr4.145290 is based on predictions only and has not been experimentally confirmed with cDNA (TAIR11).

An Arabidopsis line harbouring a T‐DNA insert in At4G17490, encoding *ERF6*, contained an average of 28% more egg masses per plant than the corresponding wild‐type Arabidopsis plants (Figs 2A and S3C, see Supporting Information). The mRNA level of the *ERF6* transcript in this mutant line (*erf6‐1*) was significantly reduced, suggesting that the increase in susceptibility is indeed caused by *ERF6* (Fig S2C). Unfortunately, for the neighbouring locus At4G17500, encoding *ETHYLENE RESPONSE FACTOR 1a *(*ERF1a*), no homozygous T‐DNA insertion line was available. It should be noted that *ERF1a* is sometimes also referred to as *AtERF1* and is distinct from *ERF1* (At3G23240), which is located on chromosome 3 (Nakano et al., 2006).

Two T‐DNA insertion lines for genes located to the right of marker Chr4.145290 (i.e. At4G17510 and At4G17530; Fig. 1) did not show a significantly different number of egg masses per plant when compared with wild‐type Arabidopsis plants (Fig. 2C,D). The homozygous T‐DNA insert at the 3’‐end of the predicted coding sequence of At4G17510, encoding *UBIQUITIN C‐TERMINAL HYDROLASE 3* (*UCH3*), interrupted the open reading frame, but did not significantly alter the amplification of mRNA upstream of the insert (Fig. S3D). The T‐DNA insert in locus At4G17530, encoding *RAB GTPASE HOMOLOG 1C* (*RAB1C*), reduced the transcript level of this gene (Fig. S3E). Seeds of Arabidopsis lines harbouring a T‐DNA insert in At4G17520, encoding *HYALURONAN/mRNA BINDING FAMILY PROTEIN* (*HLN*), showed an aberrant germination phenotype and could therefore not be used in nematode bioassays. We therefore concluded that, for QTL13, only *ERF6* is likely to be involved in the reproductive success of *M. incognita* in Arabidopsis. Importantly, the number of root tips and the total root length of seedlings of the *erf6‐1 *T‐DNA mutant line were not significantly different from those of wild‐type Arabidopsis plants (Fig. S4, see Supporting Information). The increase in reproductive success of *M. incognita* in this mutant can therefore not be attributed to significant changes in root morphology.

**Figure 3:**
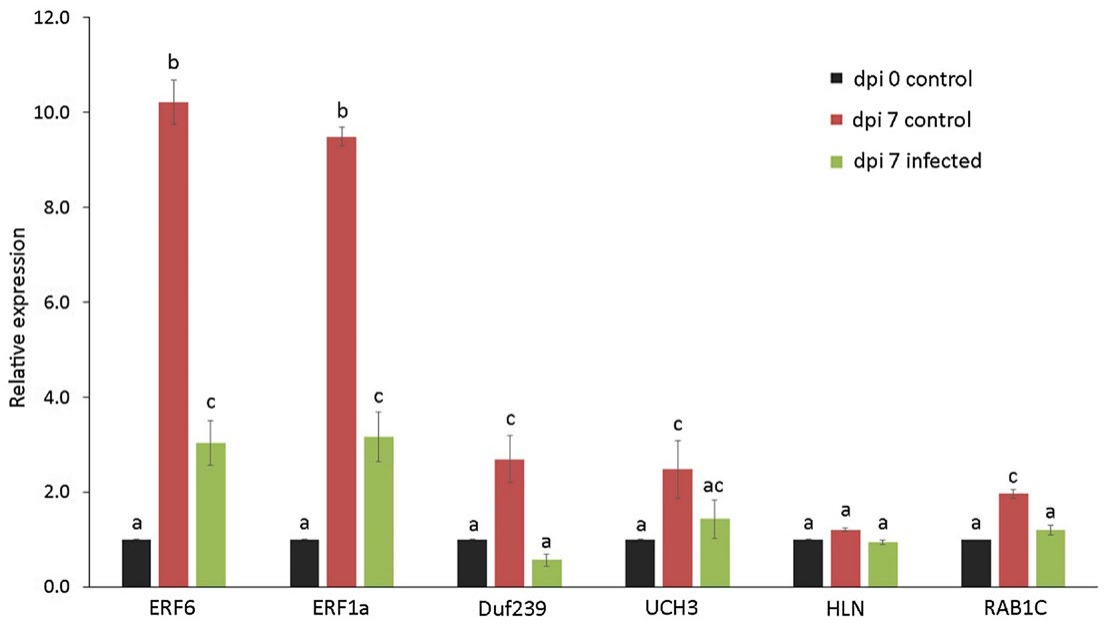
Relative expression of *ERF6*, *ERF1a*, *Duf239*, *UCH3*, *HLN* and *RAB1C* in roots of wild‐type Arabidopsis Col‐0 plants on infection with *Meloidogyne incognita*. Data reflect the gene expression levels determined by quantitative reverse transcription‐polymerase chain reaction (qRT‐PCR) in whole roots collected at the time of inoculation with *M. incognita* (dpi 0 control), in whole roots collected at 7 days after mock inoculation (dpi 7 control) and in whole roots collected at 7 days post‐inoculation (dpi) with *M. incognita* (dpi 7 infected). The bars represent average values relative to the expression at 0 dpi. The data were based on three independent biological samples with three technical replicates per sample. Error bars represent the standard error of the mean. Different letters indicate statistically significant differences for each gene of interest separately as determined by analysis of variance (ANOVA) with *post‐hoc* Tukey’s honestly significant difference (HSD) test (*P* < 0.05). [Colour figure can be viewed at wileyonlinelibrary.com]

To find further support for the involvement of QTL13 in nematode infections, we analysed the expression of the six genes by quantitative reverse transcription‐polymerase chain reaction (qRT‐PCR) in whole Arabidopsis roots at 7 days post‐inoculation (dpi) with *M. incognita*. We also included samples of wild‐type Arabidopsis seedlings at the time of inoculation to assess whether these genes undergo a significant developmental regulation in roots in the absence of root‐knot nematodes. Both *ERF6* and *ERF1a* were strongly up‐regulated in non‐infected plants during the first 7 days after mock inoculation, but were significantly repressed in plants infected with *M. incognita* (Fig. 3). The relative expression of *DUF239* followed largely the same pattern over the three samples. The relative expression of *UCH3* and *HLN* was not significantly different between infected and non‐infected Arabidopsis roots. The relative expression of *RAB1C* showed a small, but significant, difference between infected and non‐infected Arabidopsis roots at 7 dpi.

### ERF6 affects the expression of abiotic stress response genes

Our data showed that the transcription factor ERF6 most probably regulates the susceptibility of Arabidopsis to *M. incognita* infection. To gain more insights into the genes regulated by ERF6, we studied changes in the transcriptome of whole roots of the *erf6‐1* mutant and the corresponding wild‐type Arabidopsis at the time of inoculation (0 dpi) and at 7 dpi with *M. incognita* using gene expression microarrays*.* We also included mock‐inoculated seedlings to monitor developmentally regulated genes in both the *erf6‐1* mutant and wild‐type Arabidopsis plants. We found no differentially expressed genes between the *erf6‐1* mutant and the wild‐type plants at the time of inoculation and 7 days later in non‐infected plants (*q* < 0.05). This showed that *ERF6* does not have a significant impact on global gene expression in developing roots of non‐infected Arabidopsis seedlings in the first 7 days after inoculation.

**Figure 4:**
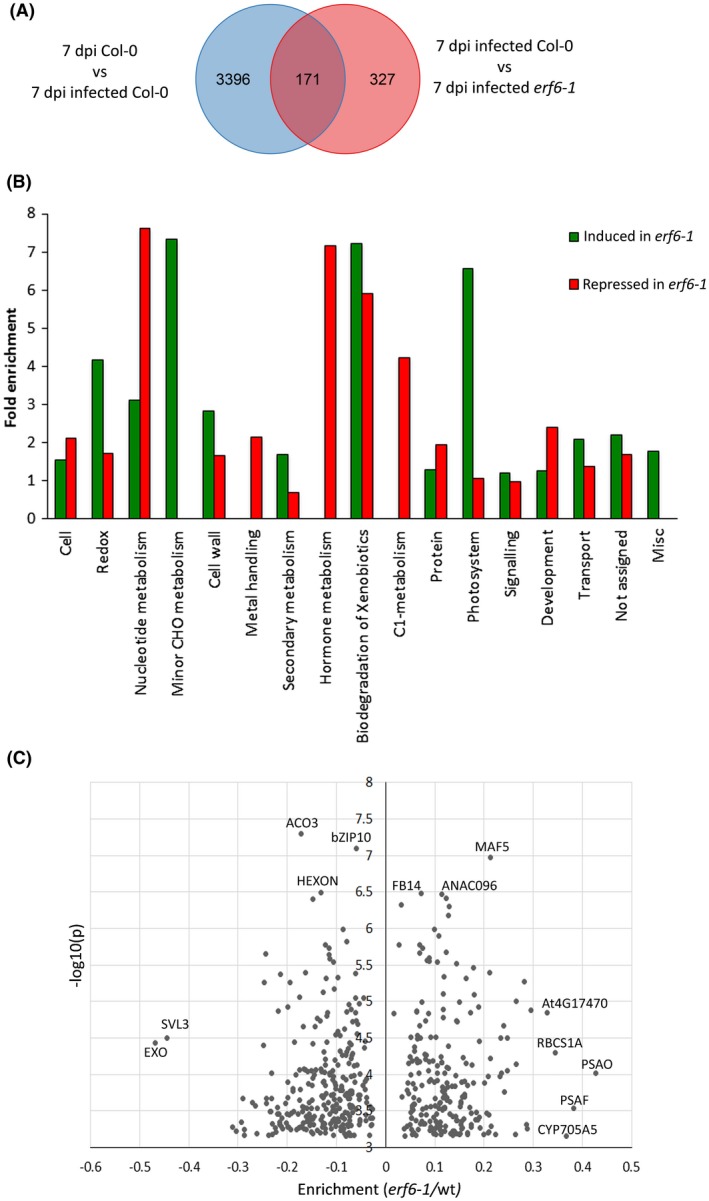
Differential expression of genes in wild‐type Arabidopsis Col‐0 and *erf6‐1* mutant at 7 days post‐inoculation (dpi) with *Meloidogyne incognita*. (A) Venn diagram showing the number of differentially expressed genes on inoculation with *M. incognita* in wild‐type Arabidopsis plants (7 dpi mock Col‐0 vs. 7 dpi infected Col‐0) and *erf6‐1* mutant plants (7 dpi infected Col‐0 vs. 7 dpi infected *erf6‐1*). (B) Functional classification of 498 differentially expressed genes between *erf6‐1* and wild‐type Arabidopsis plants at 7 dpi of *M. incognita* into MapMan bins. On the *x*‐axis are the main BINs to which the differentially expressed genes belong. The *y*‐axis represents the fold enrichment of the number of genes within a bin. (C) Volcano plot of the 498 differentially expressed genes in nematode‐infected roots of *erf6‐1* mutant plants and wild‐type Arabidopsis Col‐0 plants. The *x*‐axis reflects the enrichment in mean normalized expression levels, and the *y*‐axis indicates the level of significance [–log_10_(*P*)]. Genes strongly regulated in *erf6‐1* mutant plants (mean normalized enrichment of <−0.30 or >0.30) and with high statistical support [–log_10_(*P*) > 6.5] are labelled with their abbreviated gene names. As expected, *ERF6* is by far the most down‐regulated gene in *erf6‐1* mutant plants (mean normalized enrichment factor of −1.97), but is not shown in the plot. [Colour figure can be viewed at wileyonlinelibrary.com]

As expected, *M. incognita* had a profound effect on the transcriptome in roots of wild‐type Arabidopsis at 7 dpi. We found 3567 differentially expressed genes in the comparison between infected and non‐infected roots of wild‐type Arabidopsis. Of this set, 171 genes were differentially expressed between nematode‐infected roots of the *erf6‐1* mutant and wild‐type Arabidopsis. Lastly, we identified 327 genes that were differentially expressed in nematode‐infected roots of the *erf6‐1* mutant, but not in infected roots of wild‐type Arabidopsis plants. In conclusion, ERF6 has a significant impact on the expression of 498 genes in nematode‐infected roots of Arabidopsis seedlings at 7 dpi (Fig. 4A; Table S4, see Supporting Information).

To determine which cellular processes are most probably affected by ERF6 in nematode‐infected roots of Arabidopsis, we first performed an enrichment analysis using MapMan bins (Thimm et al., 2004). The set of 498 genes differentially regulated by ERF6 in nematode‐infected roots of Arabidopsis was enriched for processes referred to as redox, nucleotide metabolism, minor carbohydrate metabolism, biodegradation of xenobiotics, C1 metabolism, photosystem and hormone metabolism (fold enrichment > 2.5) (Fig. 4B). MapMan enrichment analysis uses genome annotation terms, but does not include the actual fold changes or statistical significances of the differences in transcripts per gene. We therefore focused further on genes that stood out because of an exceptionally large change in expression level (i.e. mean normalized change in probe intensity of <−0.3 or >0.3), because of high statistical support of the changes (−log_10_ > 6.5), or both (Fig. 4C). The application of these criteria resulted in a list of 15 genes with an overrepresentation of transcription factors, photosystem components and mediators of abiotic stress responses (Table 2).

**Table 2:** The 15 genes most strongly or significantly regulated in roots of *erf6‐1 *and wild‐type Col‐0 plants at 7 days post‐inoculation with *Meloidogyne incognita*.

Gene ID*	Relative expression†	−log_10_(*P*)‡	Bonferroni	Description	Stress response§
AT1G12010	–0.17259	7.29	0.0009	1‐Aminocyclopropane‐1‐carboxylate oxidase 3 domain (ACO3)	Oxidative stress (Sewelam et al., 2013; Wang et al., 2013); osmotic stress (Dubois et al., 2015)
AT4G02640	–0.05964	7.10	0.0009	Basic leucine zipper transcription factor (bZIP10)	Oxidative stress (Kaminaka et al., 2006)
AT5G65080	0.21294	6.97	0.0010	MADS‐box transcription factor (MAF5)	Unknown
AT5G54850	–0.13181	6.49	0.0016	Hexon like	Unknown
AT1G20800	0.07244	6.48	0.0016	F‐box family protein (FB14)	Unknown
AT5G46590	0.11422	6.47	0.0016	NAC transcription factor ANAC096	Dehydration and osmotic stress (Xu et al., 2013)
AT4G08950	–0.46799	4.43	0.0433	Phosphate‐responsive 1 family protein (EXO)	Salt stress (Renault et al., 2013); oxidative stress (Charron et al., 2008)
AT3G20520	–0.44503	4.49	0.0114	Glycerophosphodiester phosphodiesterase‐like family protein (SVL3)	Salt stress (Ma et al., 2006)
AT2G29470	–0.31112	3.28	0.0432	Glutathione *S*‐transferase tau 3 (GSTU3)	Toxic catabolic process; oxidative stress (Loeffler et al., 2005)
AT1G70260	–0.30337	3.22	0.0465	Nodulin MtN21/EamA‐like transporter family protein (RTP1)	Oxidative stress (Pan et al., 2016)
AT4G17470	0.32912	4.85	0.0076	α/β‐Hydrolases superfamily protein	Unknown
AT1G67090	0.34508	4.29	0.0148	Ribulose bisphosphate carboxylase small chain 1A (RBCS1A)	Unknown (chloroplast)
AT5G47990	0.36753	3.16	0.0497	Cytochrome P450, family 705, subfamily A, polypeptide 5 (CYP705A5)	Unknown (chloroplast)
AT1G31330	0.38302	3.54	0.0331	Photosystem I subunit F (PSAF)	Unknown (chloroplast)
AT1G08380	0.42666	4.01	0.0196	Photosystem I subunit O (PSAO)	Unknown (chloroplast)

*Genes are indicated with their locus identifier (in TAIR).

†Relative expression to the mean (log_2_ value).

‡Level of significance before [−log_10_(*P*)].

§Involvement in response to different types of stress.

**Figure 5:**
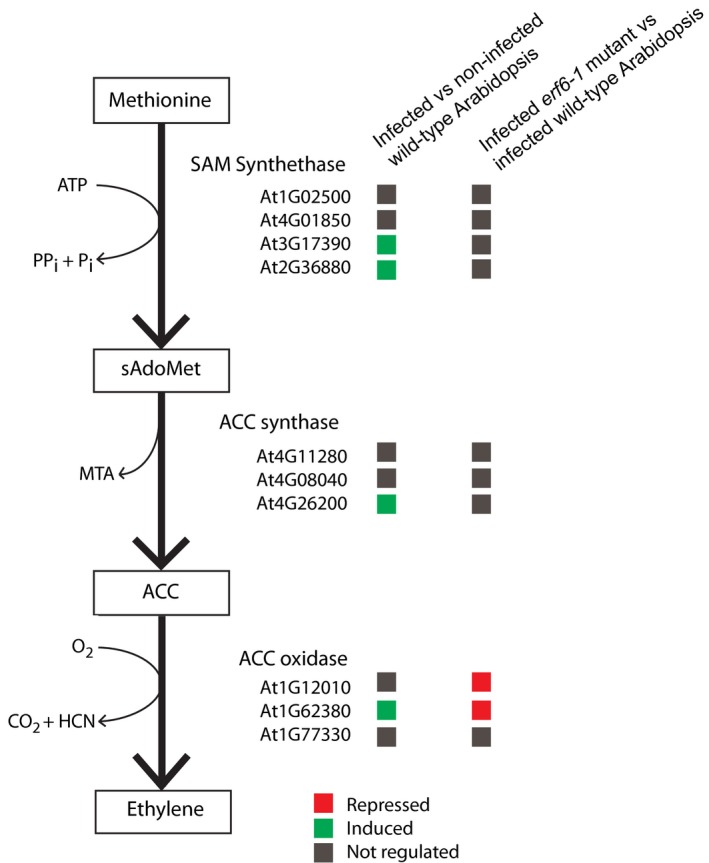
Differential regulation of genes encoding enzymes involved in the ethylene biosynthesis pathway on infection in whole roots of the *erf6‐1* mutant and wild‐type Arabidopsis plants at 7 days post‐inoculation with *Meloidogyne incognita*. The coloured boxes indicate induction (blue) or repression (red) of gene expression in a comparison between nematode‐infected and non‐infected roots of wild‐type Arabidopsis plants (left column). The boxes in the right column indicate induction or repression of gene expression in nematode‐infected roots of *erf6‐1* vs. wild‐type Arabidopsis plants. ACC, aminocyclopropane‐1‐carboxylate; ATP, adenosine triphosphate; MTA, 5’‐methylthioadenosine; Pi, adenosine monophosphate; PPi, pyrophosphate; SAM, *S*‐adenosyl‐methionine; sAdoMet, *S*‐adenosylmethionine. [Colour figure can be viewed at wileyonlinelibrary.com]

ERF6 is thought to be involved in tolerance to abiotic stress regulated by reactive oxygen species (ROS) and 1‐aminocyclopropane‐1‐carboxylic acid (ACC; Sewelam et al., 2013). Strikingly, the gene with highest statistical support for being down‐regulated in a comparison between nematode‐infected roots of the *erf6‐1* mutant line and wild‐type Arabidopsis encodes 1‐aminocyclopropane‐1‐carboxylate oxidase 3 (At2G12010; ACC oxidase 3 or ACO3), which regulates the accumulation of ACC in plant cells by its conversion into ethylene. ACC is derived from methionine and *S*‐adenosyl‐methionine (SAM) in a series of reactions catalysed by SAM synthetases and ACC synthases. In a comparison between infected and non‐infected roots of wild‐type Arabidopsis plants, we observed that nematode infection is associated with an up‐regulation of SAM synthetase 3 and 4, ACC synthase 7 (ACS7) and ACC oxidase 2 (ACO2), but not ACO3 (Fig. 5; Table S3, see Supporting Information). However, in the comparison between nematode‐infected roots of the *erf6‐1* mutant and wild‐type Arabidopsis, we observed a down‐regulation of both ACO2 and ACO3 at 7 dpi with *M. incognita *in our gene expression array data (Fig. 5).

## DISCUSSION

Recently, we have used GWA mapping to resolve the architecture of susceptibility to *M. incognita* in a population of 340 Arabidopsis natural inbred lines (Warmerdam et al., 2018). We linked the susceptibility to *M. incognita* to allelic variation in only four loci on two chromosomes of Arabidopsis, which was below our expectations. We therefore reasoned that, by using a relatively high threshold for significance (i.e. –log_10_(*P*) > 5) in our original analyses (Warmerdam et al., 2018), we might have disregarded valuable signals. Moreover, the SNPs identified in our original analysis could only explain 50% of the additive heritable phenotypic variation, providing further support for the idea that, by relaxing the stringency of our analysis, we might be able to resolve more components of the architecture of susceptibility. Here, we showed that, by accepting −log_10_(*P*) ≥ 4 as the threshold for significant associations between SNPs in Arabidopsis and the reproductive success of *M. incognita*, we could resolve all of the heritable phenotypic variation in our population of Arabidopsis lines (Table S1).

As the false discovery rate increases by lowering the threshold of significance for association mapping, we challenged the risk of pursuing false positives by further investigating QTL13. The association of susceptibility with QTL13 was based on a single SNP with a significance on the threshold value [−log_10_(*P*) = 4] and with the smallest effect size of all SNPs meeting this criterion. SNP Chr4.145290, marking QTL13, is located within the predicted coding sequence AtG17505 (*DUF239*). However, we found no further evidence to conclude that allelic variation in *DUF239* contributes to the variation in susceptibility of Arabidopsis to *M. incognita*.

Five other genes are located within the 20‐kb region around SNP Chr4.145290 and could therefore also be causally linked to the susceptibility to *M. incognita *(Kim et al., 2007 *)*. However, we noted that Chr4.145290 marks a sharp boundary between a region harbouring SNPs with relatively high −log_10_(*P*) values at one side and a region with relatively low −log_10_(*P*) values at the other (Fig. 1). This suggests that the decay in LD may not be evenly distributed around Chr4.145290, and that the three genes (i.e. *UCH13*, *HLN1* and *RAB1C*) located in the flanking region with lower statistical support are less likely to be causally linked to susceptibility (Fig. 2). Indeed, our analyses with T‐DNA mutant lines and the expression of the genes provide no further evidence that *UCH13*, *HLN1* and *RAB1C* are involved in the susceptibility of Arabidopsis to *M. incognita*.

The two remaining genes in QTL13, *ERF1a* and *ERF6*, belong to the APETALA2/ETHYLENE RESPONSE FACTOR (AP2/ERF) superfamily of transcription factors. Members of the AP2/ERF superfamily are divided into four families based on the number of AP2/ERF domain repeats. Together with 120 other relatives, ERF1a and ERF6 form the largest class of AP2/ERF transcription factors in Arabidopsis. ERF family members typically harbour only one AP2/ERF domain of about 70 amino acids, which binds DNA at an AGCCGCC motif (GCC‐box) in promoter regions of the target genes. The ERF family is further divided into 12 subgroups based on conserved motifs outside the AP2/ERF domain, some of which are involved in the activation and deactivation of the transcription factors. ERF1a belongs to subgroup XIa, whereas ERF6 is assigned to subgroup IXb. Our data showed that both ERF1a and ERF6 are strongly down‐regulated in association with nematode infections in roots of Arabidopsis seedlings at 7 dpi (Fig. 3). Unfortunately, no homozygous T‐DNA insertion mutant line was available for the *ERF1a* gene in the Arabidopsis stock centres. Therefore, we cannot exclude the possibility that allelic variation in *ERF1a* contributes to the variation in reproductive success of *M. incognita* in our population of Arabidopsis inbred lines.

The significantly higher number of egg masses of *M. incognita* in the knockout mutant line *erf6‐1*, when compared with wild‐type Arabidopsis plants, showed that ERF6 most probably functions as a susceptibility gene for *M. incognita* in Arabidopsis (Fig. 2). *ERF6* is thought to be functionally redundant with *ERF5*, which is the closest relative of *ERF6* within ERF subgroup IXb (Moffat et al., 2012; Son et al., 2012). However, our data suggest that *ERF5* and *ERF6* are not redundant in co‐regulating the susceptibility to *M. incognita *in Arabidopsis. The single *erf6‐1* mutant line, harbouring an intact *ERF5* gene, was more susceptible to nematodes. Previously, the *erf6‐1* mutant has been shown to be more susceptible to the virulent *Pseudomonas syringae *DC3000 strain when compared with wild‐type Arabidopsis plants, which also points to the functional diversification of both genes (Son et al., 2012). Furthermore, if *ERF5* and *ERF6* function as redundant susceptibility genes for *M. incognita *infections in Arabidopsis, we would probably have found significant associations linked to *ERF5* in our GWA analyses. We observed no such association between the number of egg masses of *M. incognita* per plant and SNPs in LD with *ERF5* on chromosome 5. We therefore conclude that allelic variation in *ERF6*, but not in *ERF5*, may contribute to the quantitative variation in reproductive success of *M. incognita* in our population of Arabidopsis inbred lines.

The transcriptional activity of ERF6 in Arabidopsis is regulated by MITOGEN‐ACTIVATED PROTEIN KINASE3 (MPK3)/MPK6 in response to biotic and abiotic stress. MPK3/MPK6‐mediated phosphorylation stabilizes ERF6 and enables its targeting to the nucleus, where it binds to the GCC‐box in promoters of target genes. The GCC‐box is found in promoters of pathogenesis‐related genes typically associated with ethylene/jasmonic acid‐dependent responses, and is a common target of multiple ERF transcription factors belonging to different subgroups (Fujimoto et al., 2000; Song et al., 2005). It is therefore possible that the enhanced susceptibility of the *erf6‐1* mutant line is caused by weakened plant defence responses. However, our MapMan enrichment analysis of genes differentially expressed in nematode‐infected roots of the *erf6‐1* mutant vs. wild‐type Arabidopsis does not point to alterations in responses to biotic stresses (Fig. 4B). Furthermore, the expression of specific pathogenesis‐related genes known to be either repressed or activated by ERF transcription factors (e.g. *PR1* [At2G14160], *PR2* [AtAt3G57260], *PR3* [At3G12520], *PR4* [At3G04720], *PDF1.1* [At1G75830] and *PDF1.2* [At5G44420]) was not significantly altered in nematode‐infected *erf6‐1* mutant plants. We therefore have no evidence to conclude that the increased susceptibility to *M. incognita* of the *erf6‐1* mutant line is related to suppressed plant defence. Nonetheless, it should be noted that we only assessed gene expression at 7 dpi and we cannot exclude the possibility that ERF6 regulates the expression of defence‐related genes at earlier and later time points.

However, many of the genes differentially regulated in the nematode‐infected roots of *erf6‐1* plants at 7 dpi are involved in responses to abiotic stresses (Fig. 4C; Table 1). This aligns well with earlier observations of ERF6 as a transcriptional regulator of genes involved in tolerance to osmotic stress (Dubois et al., 2013, 2015), ROS (Sewelam et al., 2013; Wang et al., 2013), high light treatment (Moore et al., 2014; Wang et al., 2013) and cold (Wang et al., 2013). Mild osmotic stress is thought to induce elevated cellular levels of ACC, which could, in a similar fashion to ROS, activate MPK3/MPK6 and, further downstream, ERF6 (Dubois et al., 2015; Wang et al., 2013). In addition, elevated levels of ACC are also known to induce *ERF6* expression in Arabidopsis seedlings (Son et al., 2012), but it is not known whether this requires the conversion of ACC into ethylene and whether it is ethylene that regulates the expression of ERF6. Recently, it has been shown that ACC itself can function as a signalling molecule and is able to regulate growth and development in plants independent from ethylene (reviewed in Van de Poel and Van Der Straeten, 2014). Our data suggest that ERF6 may affect the conversion of ACC into ethylene in nematode‐infected roots by regulating the expression of ACO2 and ACO3 (Fig. 5; Table 1), which could point to a key role for ethylene in ERF6‐mediated susceptibility to *M. incognita*. However, unlike several other ERF transcription factors, ERF6‐mediated expression of target genes has been shown to be independent of ethylene signalling (Meng et al., 2013). Alternative signalling pathways are therefore likely to contribute to the ERF6‐regulated susceptibility of Arabidopsis to *M. incognita*, possibly involving ACC as a signalling molecule (Dubois et al., 2015).

Recently, we have shown in a multi‐trait analysis, using the same panel of natural inbred lines of Arabidopsis, a strong positive correlation (*R* = 0.8) between SNPs significantly associated with the response to osmotic stress [i.e. increase in biomass under polyethylene glycol 8000 (PEG8000) treatment] and the reproductive success of *M. incognita* (Thoen et al., 2017). Interestingly, no such correlation was found between the susceptibility to *M. incognita* and responses to salt, drought and heat. This indicates that alleles specifically regulating tolerance to osmotic stress in Arabidopsis may also regulate the susceptibility to *M. incognita*. Permanent feeding cells induced by *M. incognita* display enormous cellular hypertrophy, which could be a response to the strongly elevated osmotic pressure in host cells caused by phloem unloading of carbohydrates and amino acids (Smant et al., 2018). Allelic variation in ERF6 may therefore affect the ability of Arabidopsis to accommodate high osmotic pressures, which could affect giant cell expansion and thereby the reproductive success of *M. incognita*.

In addition to alterations in stress response genes, we also noted a strongly enhanced expression of various components of photosystem I in nematode‐infected roots of the *erf6‐1* mutant line (e.g. PSAO, PSAF, RBCS1A; Fig. 4C). This observation is puzzling as, during the *in vitro* bioassays with *M. incognita*, the Arabidopsis roots were not exposed to light*.* Differential regulation of components involved in photosynthesis is often observed in association with nematode infections in plants, and our findings are unlikely to be an artefact caused by specific environmental factors in our experimental set‐up. Even in soil‐grown plants, nematode‐induced permanent feeding cells can harbour large numbers of chloroplast‐like organelles, but the role of these structures is not well understood (Golinowski et al., 1996; Kyndt et al., 2012).

In conclusion, in this study, we have shown that, by accepting a lower threshold for significance and higher false discovery rate in GWA mapping, we were able to resolve all of the heritable variation in susceptibility to *M. incognita* in our population of Arabidopsis lines. In total, we identified 19 QTLs significantly associated with the reproductive rate of *M. incognita* on Arabidopsis, some of which may still be false positives. However, by focusing on an SNP marker Chr4.145290 that we initially considered to be a probable false positive, we gained insight into the role of ERF6 as a regulator of abiotic stress responses in plant–nematode interactions.

## EXPERIMENTAL PROCEDURES

### Plant material

The following homozygous Arabidopsis T‐DNA insertion mutant lines were obtained from the Nottingham *Arabidopsis* Stock Centre (Alonso et al., 2003): SALK_087356C with T‐DNA insert in At4G17490 (*erf6‐1*); SALK_059419C with T‐DNA insert in At4G174505 (*duf239‐1*); SALK_087258C with T‐DNA insert in At4G147510 (*uch3‐1*); SALK_129052C with T‐DNA insert in At4G17520 (*hln‐1*); and SALK_104999C with T‐DNA insert in At4G17530 (*rab1c‐1*). The T‐DNA insert in all mutant lines interrupted the (predicted) open reading frames in the coding sequences of the genes. The T‐DNA insertion lines were all generated in the background of Col‐0 (N60000), which was used as wild‐type Arabidopsis throughout this study.

The presence and homozygosity of the T‐DNA insert in the mutant lines were checked by PCR on genomic DNA isolated from leaf material of 12 seedlings (Warmerdam et al., 2018). To determine whether the wild‐type allele or the T‐DNA insert was present in the mutants, we used different combinations of gene‐specific forward and reverse primers and combinations of gene‐specific primer and T‐DNA‐insert‐specific Lbl3.1 primer (Table S2, see Supporting Information; Alonso et al., 2003). For PCR, we used the following conditions: 10 min at 94 ^o^C, 35 cycles of 30 s at 94 ^o^C, 1.5 min at 60 ^o^C and 1 min at 72 ^o^C, and a final incubation of 10 min at 72 ^o^C. The PCR amplification products were analysed by agarose gel electrophoresis.

We also checked the expression of the affected gene by the T‐DNA insertion line by qRT‐PCR. Total RNA was isolated from whole roots of 12 14‐day‐old plants of the specific T‐DNA insertion mutant, as described below for the microarray analysis. First‐strand cDNA was synthesized from total RNA using a Superscript III First‐Strand synthesis system according to the manufacturer’s standard protocol (Invitrogen, Leiden, The Netherlands). cDNA samples were used as template in qPCR with the Absolute SYBR Green Fluorescein mix (Thermo Fisher Scientific, Leiden , The Netherlands). cDNA matching *Arabidopsis thaliana* elongation factor 1α (At5G60390) was amplified as a reference for gene constitutive expression (Czechowski et al., 2004). To quantify the expression level for the gene of interest, we used gene‐specific forward and reverse primers (Table S2). For PCR, we used the following conditions: 15 min at 95 ^o^C, 40 cycles of 30 s at 95 °C, 30 s at 62 °C and 30 s at 72 °C, and a final incubation of 5 min at 72 °C. The relative expression ratio between the gene of interest and the reference gene was calculated as described elsewhere (Pfaffl, 2001).

#### Nematode bioassays

Eggs of *M. incognita* were obtained by treating tomato roots infected with *M. incognita* (strain ‘Morelos’ from INRA, Sophia Antipolis, France) with 0.05% v/v NaOCl for 3 min. Roots were rinsed with tap water and the eggs were collected on a 25‐μm sieve. Next, the eggs were incubated in a solution of 2.4 mm NaN_3_ for 20 min whilst shaking. Thereafter, the eggs were rinsed with sterile tap water and incubated on a 25‐μm sieve in a solution of 1.5 mg/mL gentamycin and 0.05 mg/mL nystatin in the dark at room temperature. Hatched juveniles were collected after 4 days and surface sterilized using 0.16 mm HgCl_2_, 0.49 mm NaN_3_ and 0.002% v/v Triton X‐100 for 10 min. After surface sterilization, the juveniles were rinsed three times with sterile tap water and transferred to 0.7% Gelrite solution (Duchefa Biochemie, Haarlem, The Netherlands).

To test the susceptibility of Arabidopsis seedlings, seeds were vapour sterilized (with 7.1 m NaOCl and 1% HCl in tap water) for 5 h and transferred to a six‐well cell culture plate containing Murashige and Skoog (MS) medium [MS with vitamins 4.7 g/L (Duchefa Biochemie), 58.5 mm sucrose and 5 g/L Gelrite]. The six‐well plates were first incubated in the dark at 4 ºC for 3 days. Thereafter, the seeds were allowed to germinate at 21 ºC under 16 h light/8 h dark conditions in a growth cabinet for a further 7 days. Individual 1‐week‐old seedlings were subsequently transferred to separate wells in a new six‐well plate containing MS medium. The seedlings were incubated for a further 7 days at 21 ºC under a 16 h light and 8 h dark regime. Next, individual seedlings were inoculated with 180 infective J2s of *M. incognita *per plant and incubated at 24 ºC in the dark. The number of egg masses per plant was counted at 6 weeks post‐inoculation by visually inspecting the roots with a dissection microscope. Unless indicated otherwise, the differences in counts per plants were statistically analysed using two‐way analysis of variance (ANOVA) and *post‐hoc* Tukey’s honestly significant difference (HSD) test in R (version 3.0.2, www.r-porject.org). Each Arabidopsis genotype was tested in at least three independent experiments with 10 replicates per experiment. Both genotype and experiment were used as factors to test for significance.

To collect roots of infected and non‐infected Arabidopsis seedlings for gene expression analyses with microarrays and qRT‐PCR, seeds were vapour sterilized (with 7.1 m NaOCl and 1% HCl in tap water) for 5 h and transferred to a six‐well cell culture plate containing MS medium [MS with vitamins 4.7 g/L (Duchefa Biochemie), 58.5 mm sucrose and 5 g/L Gelrite]. The six‐well plates were first incubated in the dark at 4 ºC for 3 days. Thereafter, the seeds were allowed to germinate at 21 ºC under 16 h light/8 h dark conditions in a growth cabinet for 7 days. Next, four seedlings were transferred to 12‐cm^2^ plates containing MS medium, which were placed vertically in a growth cabinet for 7 days at 21 ºC under a 16 h light and 8 h dark regime. Next, individual seedlings were inoculated with 180 infective J2s of *M. incognita *per plant and incubated horizontally at 24 ºC in the dark. Samples of 12 whole‐root systems were collected at the time of inoculation, at 7 dpi with *M. incognita*, and at 7 days after mock inoculation. The samples were stored at −80 ºC until further use.

#### Root phenotypes

Arabidopsis seedlings were allowed to germinate and grow for 14 days on MS medium as described above for the nematode bioassays. To determine the total root length and number of root tips of the seedlings, the complete plants were transferred from the media onto a plastic tray with water. Next, the leaves of the seedlings were removed and the roots were spread out over the surface of the tray. The total root length was measured using the WinRHIZO package for Arabidopsis (EPSON perfection V800, WinRHIZO pro2015, Regent Instruments Inc., Quebec, Canada). The number of root tips was counted manually based on the scan made using WinRHIZO. Differences in the total root length per seedling in centimetres and the number of root tips were statistically analysed with a two‐way ANOVA and *post‐hoc* Tukey’s HSD test in R (*P* < 0.05).

#### GWA mapping

GWA mapping was performed as described previously (Warmerdam et al., 2018). In short, the GWAS function from the R‐package rrBLUP was used to apply the EMMAX method for kinship‐based GWAS using REML (Kang et al., 2010; Yu and Buckler, 2006). The kinship matrix was calculated using the A.mat function (Endelman, 2011), employing the 214 051 SNPs of the panel as input (Thoen et al., 2017). Linkage was calculated as *R* ^2^ (from the Pearson correlation) between marker pairs in *R* (version 3.3.3, x64).

#### Heritability estimations

The narrow‐sense heritability was estimated using EMMA (Kang et al., 2008; Rockman et al., 2010). We used the kinship matrix (minimum allele frequency > 5%) of the strains to estimate the genotypic variation (*V*_G_) and the residual variance (*V*_E_) using REML. These were used to calculate the narrow‐sense heritability as:

$$h^{2}=\frac{V_{\text{G}}}{V_{\text{G}}+V_{\text{E}}}$$

where *h* ^2^ is the narrow‐sense heritability. In order to determine the amount of heritable variation explained by the set of SNPs detected, the inclusion threshold was lowered from –log_10_(*P*) = 6.1 to −log_10_(*P*) = 4.0 in steps of 0.1. For each set of SNPs, an additive ANOVA model was constructed to determine the phenotypic variation captured by the set of SNPs. As a measure of model complexity vs. explanatory power, the Bayesian information criterion (BIC) was calculated using the BIC function in R.

#### RNA isolation

Expression analysis for the gene of interest was performed on the stored root samples produced during the nematode infection study. Whole‐root systems were cut from aerial parts of the seedlings and snap frozen in liquid nitrogen. Total RNA was isolated from whole roots of 12 14‐day‐old plants of *erf6‐1*, *duf239‐1*, *uch3‐1*, *rab1c‐1* and Col‐0 wild‐type. The frozen root systems were homogenized using TissueLyser (Qiagen, Venlo, The Netherlands) twice for 30 s. Total RNA was extracted from 100 mg of the homogenate with the Maxwell Plant RNA kit (Promega Corporation, Leiden, The Netherlands) using the Maxwell 16 Robot (Promega Corporation), according to the manufacturer’s protocol. A DNAse treatment was included in the Maxwell Robot processing. The amount of total RNA per sample was determined by an ND‐1000 spectrophotometer (Isogen Life Science, Utrecht, The Netherlands).

#### qRT‐PCR

For qRT‐PCR, first‐strand cDNA was synthesized from total RNA using a Superscript III First‐Strand synthesis system (Invitrogen) according to the manufacturer’s protocol. Samples were analysed by qPCR using Absolute SYBR Green Fluorescein mix (Thermo Fisher Scientific). cDNA matching *Arabidopsis thaliana* elongation factor 1α was amplified as a reference for constitutive expression using the primers indicated in Table S2 (Czechowski et al., 2004). To quantify the expression level for the gene of interest, specific gene primers were used, which were all checked for amplification efficiency (Table S2). For qRT‐PCR, 5 ng of cDNA was used with the following conditions: 15 min at 95 ºC, 40 cycles of 30 s at 95 ºC, 30 s at 62 ºC and 30 s at 72 ºC, and a final incubation of 5 min at 72 ºC. The relative expression ratio between the gene of interest and the reference gene was calculated as described elsewhere (Pfaffl, 2001). The relative expression ratio was statistically analysed for significance with a two‐way ANOVA and *post‐hoc* Tukey’s HSD test in *R* (*P* < 0.05).

#### Microarray analysis

For microarray analysis, approximately 825 ng of total RNA of each sample was used for gene expression analysis on an *Arabidopsis* V4 Gene Expression Microarray (4 × 44K, Agilent Technologies, Santa Clara, California, United States). The probes were re‐blasted against TAIR11 using the BLASTN function of the command line blast tool (version 2.6.0, win64). The default settings were used and the top‐hit was used as probe annotation. Probes with multiple hits were censored (Camacho et al., 2009). Total RNA was fluorescently labelled for two‐colour microarray analysis and subsequently used for hybridization to the probes on the slides, according to the manufacturer’s protocol (QuickAmp Protocol, Agilent Technologies). Binding of fluorescent RNA to the probes on the microarray was measured with a High‐Resolution C Microarray Scanner and Feature Extraction Software (Agilent). Four independent replicates were made of roots of Col‐0 at the time of inoculation (0 dpi), Col‐0 at 7 dpi and *erf6‐1* at 7 dpi. Three independent replicates were made of roots of Col‐0 at 7 dpi, *erf6‐1* at the time of inoculation (0 dpi) and *erf6‐1* at 7 dpi. All microarray data were deposited in the ArrayExpress database at EMBL‐EBI (www.ebi.ac.uk/arrayexpress) under accession number E‐MTAB‐6711.

After scanning the microarrays, the spot intensities were not background corrected prior to analysis (Zahurak et al., 2007). Gene expression profiles were normalized using the Loess method (within‐array normalization) and the quantile method (between‐array normalization) (Smyth and Speed, 2003). The normalized intensities were log_2_‐transformed for further analysis.

A linear model was used to identify differentially expressed genes in a side‐by‐side comparison. The following treatments were compared: uninfected roots of Col‐0 vs. *erf6‐1* plants at the time of inoculation; uninfected roots of Col‐0 vs. *erf6‐1* plants 7 days after mock inoculation; infected roots of Col‐0 vs. *erf6‐1* plants at 7 dpi; and uninfected roots vs. infected roots of Col‐0 plants at 7 days after the time of inoculation.

We used the linear model:

*E_i_* = *T_i_* + error

where the log_2_‐normalized expression (*E*) of spot *i* (*i* = 1, 2, ..., 45 220) was explained over treatment (*T*). Afterwards, the significances obtained were corrected for multiple testing using the false discovery rate procedure in P adjust, obtaining *q* values (Benjamini and Hochberg, 1995).

Enrichments were calculated using a hypergeometric test, as provided in ‘R’. The following databases used were mined from TAIR11 (Berardini et al., 2015; Lamesch et al., 2012): Gene ontology, Gene ontology slim, gene classes, phenotypes. Furthermore, the MapMan gene ontology database (based on TAIR10) was used (Thimm et al., 2004). Enrichments were performed on gene annotations, not on spots (e.g. a gene covered by multiple spots was counted as one). Gene groups were filtered as follows: the group needed to consist of at least three genes and the overlap should be two at a minimum.

## Supporting information


**Fig. S1** ****Manhattan plot of associations between 199 525 single nucleotide polymorphisms (SNPs) and the number of egg masses per plant of *Meloidogyne incognita* in Arabidopsis at 6 weeks post‐inoculation. The broken horizontal line indicates the threshold for significance in the genome‐wide association (GWA) mapping set at –log_10_(*P*) = 4. Green dots indicate the positions of 36 significantly associated SNPs, nine of which are overlapping and are visualized by the darker green colour of the dot. The rectangles represent the five chromosomes of Arabidopsis.

**Fig. S2** ****Linkage between 36 single nucleotide polymorphisms (SNPs) in the Arabidopsis genome significantly associated with the number of egg masses of *Meloidogyne incognita* per plant. The linkage was calculated using the *R* ^2^ method and expressed as the level of correlation in shades of purple. Presented here is a pairwise comparison between all 36 SNPs as indicated by their positional marker. The grey rectangles present the five chromosomes of Arabidopsis.

**Fig. S3** ****Confirmation of T‐DNA insert in lines *erf6‐1*, *duf239‐1*, *uch3‐1* and *rab1c‐1* and the presence of actin in wild‐type Arabidopsis Col‐0 with polymerase chain reaction (PCR). (A) Allele‐specific PCRs on genomic DNA isolated from each Arabidopsis mutant line. PCR amplification products using primer combinations for only the wild‐type gene allele (P1) and for the wild‐type allele harbouring a T‐DNA insert (P2). PCR amplification product using primer combination specific for actin in genomic DNA isolated from wild‐type Arabidopsis Col‐0. (B–E) Relative gene expression of the genes harbouring the T‐DNA insert in the mutant lines when compared with the wild‐type Arabidopsis Col‐0 using quantitative reverse transcription‐polymerase chain reaction (RT‐PCR) on 14‐day‐old seedlings. (B) Relative gene expression of *DUF239* in *duf239‐1*. (C) Relative gene expression of *ERF6* in *erf6‐1*. (D) Relative gene expression of *UCH‐3* in *uch3‐1*. (E) Relative gene expression of *Rab1C* in *rab1c‐1*. Data in (B–E) were generated with three independent biological replicates with three technical replicates each.

**Fig. S4** ****Total root length and number of root tips of wild‐type Arabidopsis plants and *erf6‐1* mutant line. (A) Number of root tips per plant of 14‐day‐old seedlings. (B) Total root length of 14‐day‐old seedlings. Data were analysed with analysis of variance (ANOVA) and *post‐hoc* Tukey’s honestly significant difference (HSD) test for significant differences (*P* < 0.05; *n* > 12).

**Table S1** ****Percentage of heritable variation explained for different significance thresholds of –log_10_(*P*) with corresponding numbers of significantly associated single nucleotide polymorphisms (SNPs).

**Table S2** ****Primers used for quantitative reverse transcription‐polymerase chain reaction (qRT‐PCR) to assess gene expression levels and T‐DNA insert in homozygous Arabidopsis lines. Identifier indicates the gene target with PCR with matching forward and reverse primers. Primer efficiency is expressed as a percentage of the product amplification.

**Table S3** ****Correspondence between quantitative reverse transcription‐polymerase chain reaction (qRT‐PCR) and microarray data of gene expression levels in wild‐type Arabidopsis seedlings at 7 days post‐inoculation with *Meloidogyne incognita* and in mock‐inoculated seedlings.

**Table S4** ****Genes differentially regulated between nematode‐infected roots of the *erf6‐1* mutant and wild‐type Arabidopsis seedlings at 7 days post‐inoculation with *Meloidogyne incognita*.
