# Supplementary material for: Mediator of tolerance to abiotic stress ERF6 regulates susceptibility of Arabidopsis to Meloidogyne incognita
Source: Mol Plant Pathol. 2018 Oct 24;20(1):137–52. doi: 10.1111/mpp.12745 (PMC6430479; doi:10.1111/mpp.12745)
Supplement: Supplementary file 1 — Fig. S1 Manhattan plot of associations between 199 525 single nucleotide polymorphisms (SNPs) and the number of egg masses per plant of Meloidogyne incognita in Arabidopsis at 6 weeks post‐inoculation. The broken horizontal line indicates the threshold for significance in the genome‐wide association (GWA) mapping set at –log10(P) = 4. Green dots indicate the positions of 36 significantly associated SNPs, nine of which are overlapping and are visualized by the darker green colour of the dot. The rectangles represent the five chromosomes of Arabidopsis. [file MPP-20-137-s001.docx]

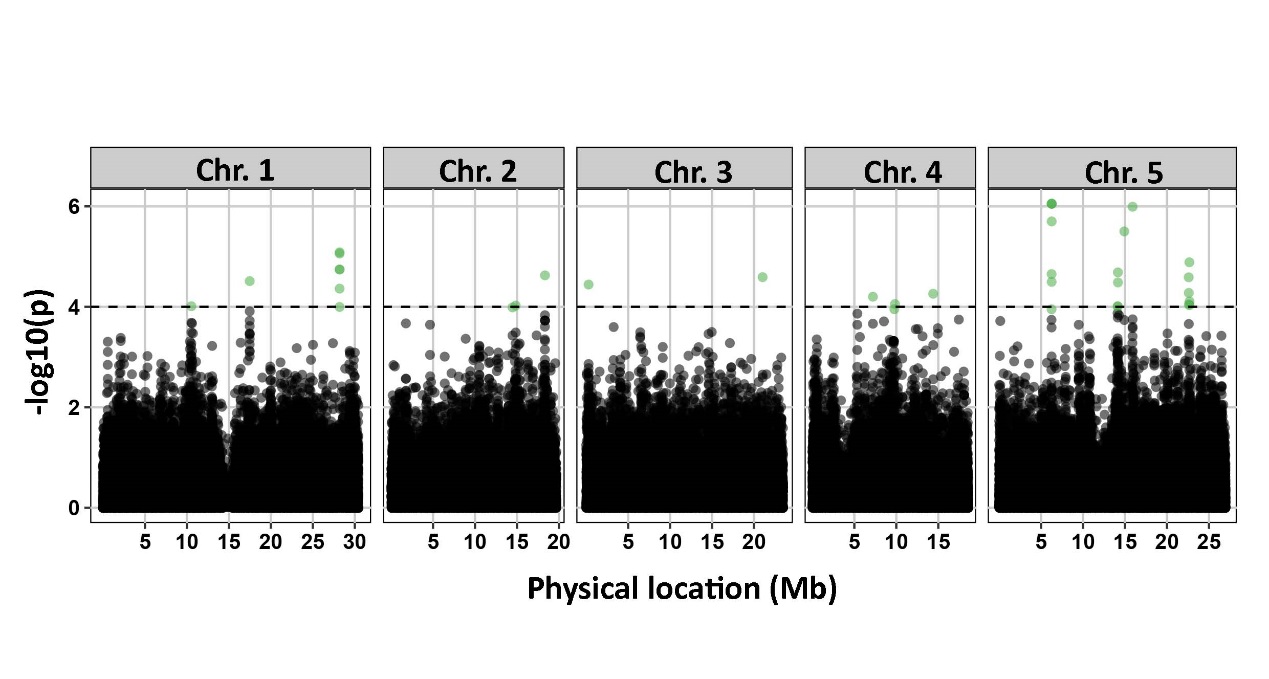


**Fig. S1.** Manhattan plot of associations between 199,525 SNPs and the number of egg masses per plant of *M. incognita* in Arabidopsis at six weeks after inoculation. Dashed horizontal line indicates threshold for significance in GWA mapping set at –log10(P) = 4. Green dots indicate the positions of 36 significantly associated SNPs, nine of which are overlapping and are visualized by the darker green color of the dot. The rectangles represent the five chromosomes of Arabidopsis.
