# Supplementary material for: Mediator of tolerance to abiotic stress ERF6 regulates susceptibility of Arabidopsis to Meloidogyne incognita
Source: Mol Plant Pathol. 2018 Oct 24;20(1):137–52. doi: 10.1111/mpp.12745 (PMC6430479; doi:10.1111/mpp.12745)
Supplement: Supplementary file 2 — Fig. S2 Linkage between 36 single nucleotide polymorphisms (SNPs) in the Arabidopsis genome significantly associated with the number of egg masses of Meloidogyne incognita per plant. The linkage was calculated using the R 2 method and expressed as the level of correlation in shades of purple. Presented here is a pairwise comparison between all 36 SNPs as indicated by their positional marker. The grey rectangles present the five chromosomes of Arabidopsis. [file MPP-20-137-s002.docx]

**
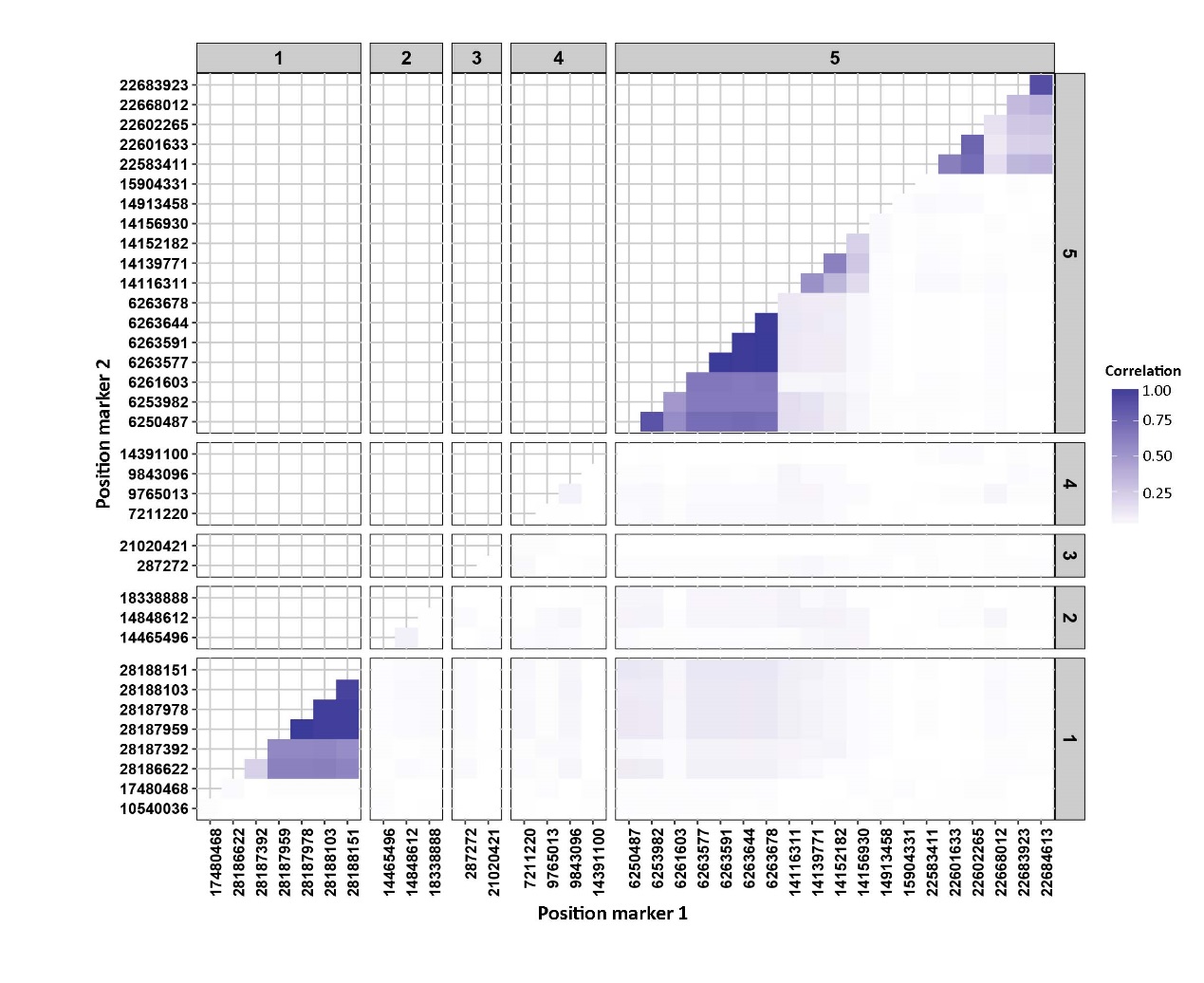
**

**Fig. S2.** Linkage between 36 SNPs in the Arabidopsis genome significantly associated with the number of egg masses of *M. incognita* per plant. The linkage was calculated using R-square method and expressed as level of correlation in shades of purple. Presented here is the pairwise comparison between all 36 SNPs as indicated by their position marker. The grey rectangles present the five chromosomes of Arabidopsis.
