# Supplementary material for: Mediator of tolerance to abiotic stress ERF6 regulates susceptibility of Arabidopsis to Meloidogyne incognita
Source: Mol Plant Pathol. 2018 Oct 24;20(1):137–52. doi: 10.1111/mpp.12745 (PMC6430479; doi:10.1111/mpp.12745)
Supplement: Supplementary file 3 — Fig. S3 Confirmation of T‐DNA insert in lines erf6‐1, duf239‐1, uch3‐1 and rab1c‐1 and the presence of actin in wild‐type Arabidopsis Col‐0 with polymerase chain reaction (PCR). (A) Allele‐specific PCRs on genomic DNA isolated from each Arabidopsis mutant line. PCR amplification products using primer combinations for only the wild‐type gene allele (P1) and for the wild‐type allele harbouring a T‐DNA insert (P2). PCR amplification product using primer combination specific for actin in genomic DNA isolated from wild‐type Arabidopsis Col‐0. (B–E) Relative gene expression of the genes harbouring the T‐DNA insert in the mutant lines when compared with the wild‐type Arabidopsis Col‐0 using quantitative reverse transcription‐polymerase chain reaction (RT‐PCR) on 14‐day‐old seedlings. (B) Relative gene expression of DUF239 in duf239‐1. (C) Relative gene expression of ERF6 in erf6‐1. (D) Relative gene expression of UCH‐3 in uch3‐1. (E) Relative gene expression of Rab1C in rab1c‐1. Data in (B–E) were generated with three independent biological replicates with three technical replicates each. [file MPP-20-137-s003.docx]

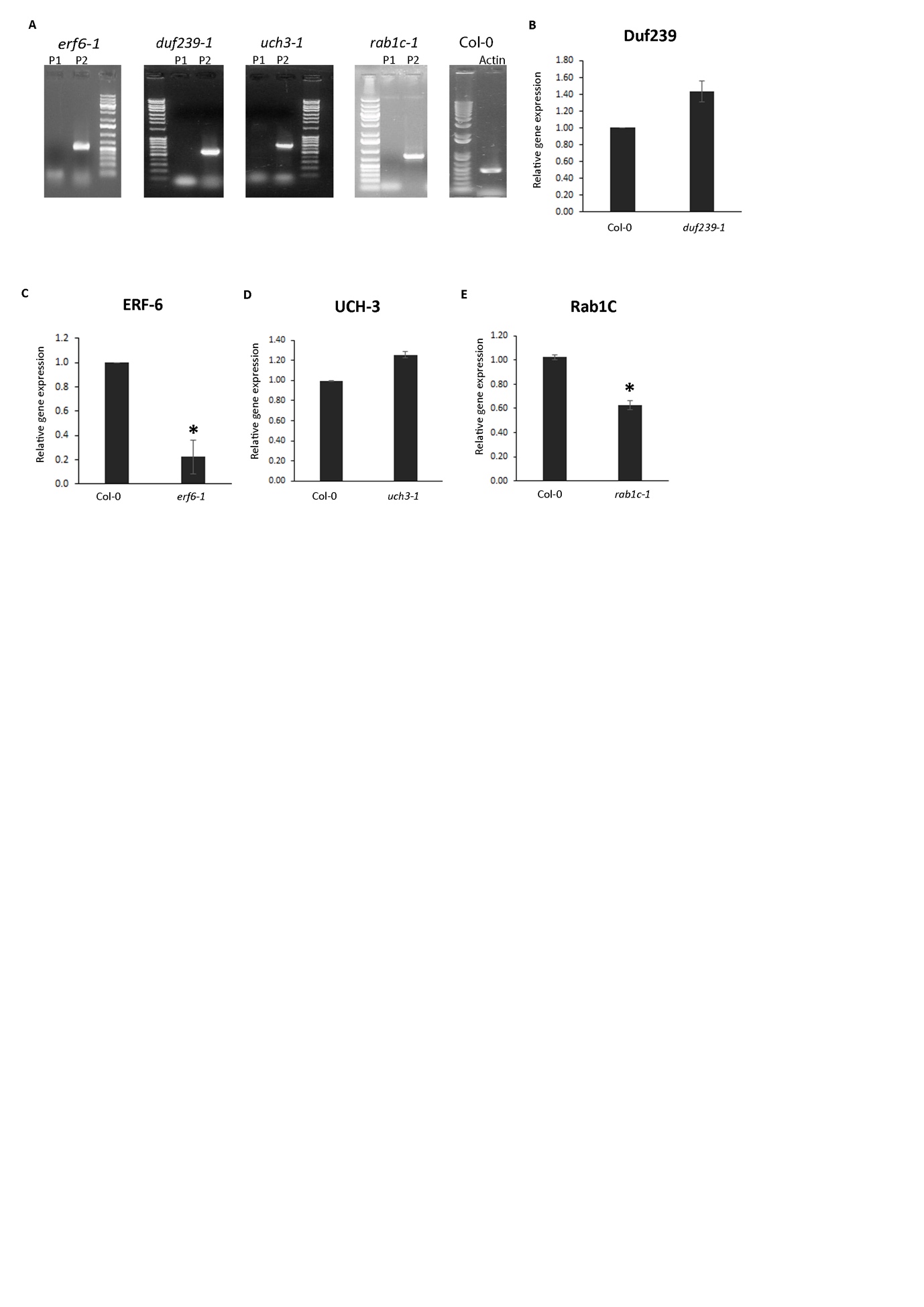


**Fig. S3.** Confirmation of T-DNA insert in lines *erf6-1*, *duf239-1*, *uch3-1* and *rab1c-1* and presence of actin in wildtype Arabidopsis Col-0 with PCR. A, allele specific PCRs on genomic DNA isolated from each Arabidopsis mutant line. PCR amplification products using primer combinations for only the wildtype gene allele (P1) and for the wildtype allele harbouring a T-DNA insert (P2). PCR amplification product using primer combination specific for actin in genomic DNA isolated from wildtype Arabidopsis Col-0. B-E, Relative gene expression of the genes harbouring the T-DNA insert in the mutant lines as compared to the wildtype Arabidopsis Col-0 using quantitative RT-PCR on 14-day old seedlings. B, represents the relative gene expression of *DUF239* in *duf239-1*. C, represents the relative gene expression of *ERF6* in *erf6-1*. D, represents the relative gene expression of *UCH-3* in *uch3-1*. E, represents the relative gene expression of *Rab1C* in *rab1c-1*. Data (B-D) was generated with three independent biological replicates with three technical replicates each.
