# Supplementary material for: Mediator of tolerance to abiotic stress ERF6 regulates susceptibility of Arabidopsis to Meloidogyne incognita
Source: Mol Plant Pathol. 2018 Oct 24;20(1):137–52. doi: 10.1111/mpp.12745 (PMC6430479; doi:10.1111/mpp.12745)
Supplement: Supplementary file 4 — Fig. S4 Total root length and number of root tips of wild‐type Arabidopsis plants and erf6‐1 mutant line. (A) Number of root tips per plant of 14‐day‐old seedlings. (B) Total root length of 14‐day‐old seedlings. Data were analysed with analysis of variance (ANOVA) and post‐hoc Tukey’s honestly significant difference (HSD) test for significant differences (P < 0.05; n > 12). [file MPP-20-137-s004.docx]

**
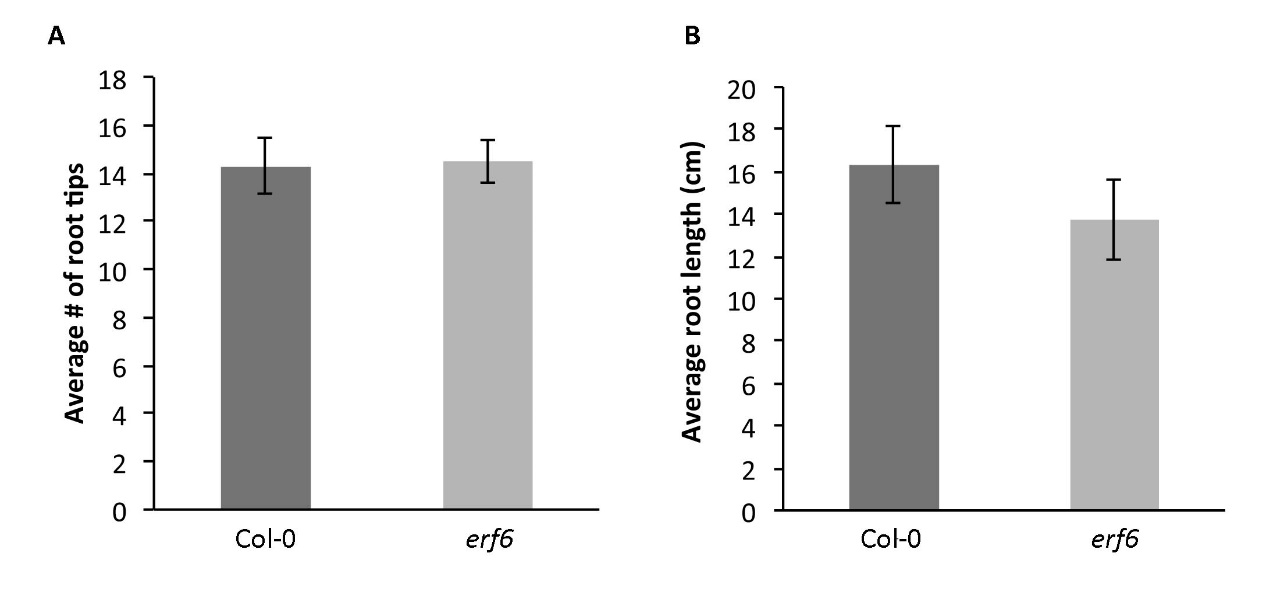
**

**Fig. S4.** Total root length and number of root tips of wildtype Arabidopsis plants and *erf6-1* mutant line. A. Number of root tips per plant of 14-day old seedlings. B. Total root length of 14-day old seedlings. Data were analysed with ANOVA and post hoc Tukey’s HSD test for significant differences (p<0.05; n>12).
