# Supplementary material for: Mediator of tolerance to abiotic stress ERF6 regulates susceptibility of Arabidopsis to Meloidogyne incognita
Source: Mol Plant Pathol. 2018 Oct 24;20(1):137–52. doi: 10.1111/mpp.12745 (PMC6430479; doi:10.1111/mpp.12745)
Supplement: Supplementary file 5 — Table S1 Percentage of heritable variation explained for different significance thresholds of –log10(P) with corresponding numbers of significantly associated single nucleotide polymorphisms (SNPs). [file MPP-20-137-s005.docx]

**Table S1.** Percentage of heritable variation explained for different significance thresholds of -Log10(p) with corresponding number of significantly associated SNPs.

| **-Log10(P)** | **Significant SNPs** | **R^2 *^** | **BIC^+^** | **% of heritable variation explained** |
| --- | --- | --- | --- | --- |
| 4 | 36 | 0.565323 | 2222.177 | 100% |
| 4.1 | 26 | 0.473539 | 2227.083 | 84% |
| 4.2 | 24 | 0.468511 | 2224.408 | 83% |
| 4.3 | 23 | 0.445424 | 2232.562 | 79% |
| 4.4 | 21 | 0.415045 | 2244.262 | 73% |
| 4.5 | 19 | 0.385783 | 2248.687 | 68% |
| 4.6 | 16 | 0.364758 | 2242.347 | 65% |
| 4.7 | 13 | 0.305151 | 2254.386 | 54% |
| 4.8 | 9 | 0.288696 | 2244.684 | 51% |
| 4.9 | 9 | 0.288696 | 2244.684 | 51% |
| 5 | 8 | 0.276729 | 2244.363 | 49% |
| 5.1 | 8 | 0.276729 | 2244.363 | 49% |
| 5.2 | 6 | 0.236795 | 2250.405 | 42% |
| 5.3 | 6 | 0.236795 | 2250.405 | 42% |
| 5.4 | 6 | 0.236795 | 2250.405 | 42% |
| 5.5 | 6 | 0.236795 | 2250.405 | 42% |
| 5.6 | 5 | 0.227295 | 2248.669 | 40% |
| 5.7 | 5 | 0.227295 | 2248.669 | 40% |
| 5.8 | 4 | 0.194646 | 2256.45 | 34% |
| 5.9 | 4 | 0.194646 | 2256.45 | 34% |
| 6 | 4 | 0.194646 | 2256.45 | 34% |
| 6.1 | 3 | 0.149554 | 2268.527 | 26% |

^*^ R^2^ reflects the amount of heritable variation explained by an additive model of the SNPS (ANOVA). ^+^ The BIC score reflects the value of the Bayesian Information Criterion, which penalizes the number of explanatory factors in a model.
