# Supplementary material for: Mediator of tolerance to abiotic stress ERF6 regulates susceptibility of Arabidopsis to Meloidogyne incognita
Source: Mol Plant Pathol. 2018 Oct 24;20(1):137–52. doi: 10.1111/mpp.12745 (PMC6430479; doi:10.1111/mpp.12745)
Supplement: Supplementary file 6 — Table S2 Primers used for quantitative reverse transcription‐polymerase chain reaction (qRT‐PCR) to assess gene expression levels and T‐DNA insert in homozygous Arabidopsis lines. Identifier indicates the gene target with PCR with matching forward and reverse primers. Primer efficiency is expressed as a percentage of the product amplification. [file MPP-20-137-s006.docx]

**Table S2.** Primers used RT-qPCR to assess gene expression levels and T-DNA insert in homozygous Arabidopsis lines. Identifier indicates the gene target with PCR with matching forward and reverse primers. Primer efficiency is expressed as percentage of the product amplification.

| **Identifier** | **Forward** | **Reverse** | **Primer efficiency** |
| --- | --- | --- | --- |
| **Expression of** |  |  |  |
| AT4G17490 (ERF6) | TCCTCCAAAATGGCTACACCA | CGATTGGTGGTGGCAGTAGT | 103 |
| AT4G17505 (ERF1A) | TCCCATCAGTGAGCTGGAGA | AATAAGGGCAAGGACCAGGC | 108 |
| AT4G17510 (Duf239) | ATCAGGGGCAAGACCAAGTC | CAGGTATTGCTCCACTCTCCT | 115 |
| AT4G17520 (UCH-3) | GGTCCTGCCAATGAAAACGG | CACCACGGTATCCACCAACA | 103 |
| AT4G17530 (Rab1C) | GGATCTAAGCCACCAACGGT | GCGAATTAAGAGGAGCAGCAG | 115 |
| At3G17390 (SAM3) | CGATGTAAGCCCCACTCCTG | ACTTATGGTGGTTGGGGTGC | 118 |
| At2G36880 (SAM4) | TGGAGCTGGAGAAGAGAGGC | TGTAACCAAGAAATCCATCCTCGT | 108 |
| At4G26200 (ACS7) | CGGAGGTCGGATGGTTTAGG | AAAACCTCCTTCGTCGGTCC | 100 |
| At1G62380 (ACO2) | TCTACGTTCGTCACCTCCCT | GTCTTTCATGGCCGTCCTGT | 107 |
| At5G60390 (EF1a) | GAGTACCACCTTTGGGACG | TTGGGTCCTTCTTGTCCACG | 107 |
| **T-DNA insert** |  |  |  |
| erf6 wildtype | TCTGAATTTGAAACCAAACCG | CGAGCATATTACATGCCATTG |  |
| erf6-1 allele | ATTTTGCCGATTTCGGAAC | CGAGCATATTACATGCCATTG |  |
| duf239 wildtype | TCTCCAGCTACCCTCCTGAAG | CTTCTGCCAGAAAGCATTCAG |  |
| duf239-1 allele | ATTTTGCCGATTTCGGAAC | CTTCTGCCAGAAAGCATTCAG |  |
| uch3 wildtype | GAATTTGGTAGGTGCATAGCG | ATCCTTTGCATGAGTTGTTGC |  |
| uch3-1 allele | ATTTTGCCGATTTCGGAAC | ATCCTTTGCATGAGTTGTTGC |  |
| hln wildtype | GACTCTGAAAGACCACGCAAG | TGGTAACCACCTCTTGGTCTG |  |
| hln-1 allele | ATTTTGCCGATTTCGGAAC | TGGTAACCACCTCTTGGTCTG |  |
| Rab1c wildtype | CTATGGATTCACGTCACACCC | ACGGAGCAAAAGGAAAAAGAG |  |
| Rab1c-1 allele | ATTTTGCCGATTTCGGAAC | ACGGAGCAAAAGGAAAAAGAG |  |
|  |  |  |  |
