# Supplementary material for: Mediator of tolerance to abiotic stress ERF6 regulates susceptibility of Arabidopsis to Meloidogyne incognita
Source: Mol Plant Pathol. 2018 Oct 24;20(1):137–52. doi: 10.1111/mpp.12745 (PMC6430479; doi:10.1111/mpp.12745)
Supplement: Supplementary file 7 — Table S3 Correspondence between quantitative reverse transcription‐polymerase chain reaction (qRT‐PCR) and microarray data of gene expression levels in wild‐type Arabidopsis seedlings at 7 days post‐inoculation with Meloidogyne incognita and in mock‐inoculated seedlings. [file MPP-20-137-s007.docx]

**Table S3.** Correspondence between RT-qPCR and micro array data of gene expression levels in wildtype Arabidopsis seedlings 7 days after inoculation with *M. incognita* and mock inoculated (7 DPI).

| **Gene** | **Fold change in expression in infected vs uninfected (RT-qPCR)** | **Relative expression infected vs uninfected (microarray)** |
| --- | --- | --- |
| At3G17390 (SAM3) | 0.902434 | +0.48 |
| At2G36880 (SAM4) | 1.600239 | +0.53 |
| At4G26200 (ACS7) | 3.574171 | +0.93 |
| At1G62380 (ACO2) | 2.061381 | +0.27 |
