# Supplementary material for: Mediator of tolerance to abiotic stress ERF6 regulates susceptibility of Arabidopsis to Meloidogyne incognita
Source: Mol Plant Pathol. 2018 Oct 24;20(1):137–52. doi: 10.1111/mpp.12745 (PMC6430479; doi:10.1111/mpp.12745)
Supplement: Supplementary file 8 — Table S4 Genes differentially regulated between nematode‐infected roots of the erf6‐1 mutant and wild‐type Arabidopsis seedlings at 7 days post‐inoculation with Meloidogyne incognita. [file MPP-20-137-s008.docx]

**Table S4.** Genes differentially regulated between nematode-infected roots of *erf6-1* mutant and wildtype Arabidopsis seedlings 7 days after inoculation with *M. incognita*.

| **Gene ID** | **-Log10(p)** | **Relative expression** | **Significance for multiple testing** |
| --- | --- | --- | --- |
| AT1G01453 | 3.985 | 0.143 | 0.020 |
| AT1G01630 | 3.327 | -0.112 | 0.042 |
| AT1G03030 | 5.994 | 0.099 | 0.003 |
| AT1G03320 | 3.709 | -0.076 | 0.028 |
| AT1G03370 | 3.916 | 0.106 | 0.022 |
| AT1G03730 | 3.724 | 0.161 | 0.027 |
| AT1G03980 | 3.656 | -0.095 | 0.029 |
| AT1G05710 | 3.704 | -0.129 | 0.028 |
| AT1G07473 | 4.245 | 0.050 | 0.016 |
| AT1G08380 | 4.013 | 0.427 | 0.020 |
| AT1G08430 | 3.350 | -0.292 | 0.041 |
| AT1G08750 | 6.408 | -0.148 | 0.002 |
| AT1G09090 | 4.132 | 0.175 | 0.018 |
| AT1G09245 | 3.682 | 0.058 | 0.028 |
| AT1G09810 | 3.354 | -0.159 | 0.040 |
| AT1G09830 | 5.322 | -0.121 | 0.004 |
| AT1G09970 | 4.966 | -0.053 | 0.007 |
| AT1G10225 | 4.263 | -0.068 | 0.015 |
| AT1G10650 | 4.207 | -0.123 | 0.016 |
| AT1G11110 | 5.894 | 0.109 | 0.003 |
| AT1G11920 | 3.530 | -0.060 | 0.033 |
| AT1G12010 | 7.294 | -0.173 | 0.001 |
| AT1G12050 | 3.257 | -0.210 | 0.045 |
| AT1G12770 | 3.909 | -0.125 | 0.022 |
| AT1G12845 | 3.370 | 0.144 | 0.040 |
| AT1G12880 | 3.420 | -0.088 | 0.038 |
| AT1G13100 | 3.574 | 0.101 | 0.032 |
| AT1G13190 | 3.834 | 0.135 | 0.024 |
| AT1G13440 | 3.455 | -0.114 | 0.036 |
| AT1G13750 | 4.165 | 0.115 | 0.017 |
| AT1G13930 | 5.560 | 0.084 | 0.003 |
| AT1G14345 | 3.279 | -0.173 | 0.043 |
| AT1G15620 | 3.313 | 0.050 | 0.042 |
| AT1G16210 | 5.821 | -0.078 | 0.003 |
| AT1G16480 | 3.774 | 0.116 | 0.025 |
| AT1G16800 | 3.484 | -0.082 | 0.035 |
| AT1G17360 | 3.347 | -0.215 | 0.041 |
| AT1G17440 | 3.186 | 0.152 | 0.049 |
| AT1G17590 | 3.404 | -0.210 | 0.039 |
| AT1G17630 | 3.477 | -0.067 | 0.036 |
| AT1G18210 | 3.619 | -0.116 | 0.030 |
| AT1G19330 | 3.284 | -0.128 | 0.043 |
| AT1G19660 | 3.376 | 0.188 | 0.040 |
| AT1G19840 | 3.198 | 0.167 | 0.048 |
| AT1G19900 | 5.000 | 0.265 | 0.007 |
| AT1G20340 | 4.897 | 0.117 | 0.008 |
| AT1G20540 | 4.438 | -0.073 | 0.012 |
| AT1G20800 | 6.484 | 0.072 | 0.002 |
| AT1G21150 | 3.361 | -0.096 | 0.040 |
| AT1G21440 | 3.498 | -0.187 | 0.035 |
| AT1G21990 | 3.309 | -0.029 | 0.042 |
| AT1G22760 | 3.849 | -0.102 | 0.024 |
| AT1G22985 | 3.479 | 0.185 | 0.036 |
| AT1G23120 | 4.216 | 0.213 | 0.016 |
| AT1G23180 | 3.772 | -0.068 | 0.025 |
| AT1G23500 | 3.620 | -0.271 | 0.030 |
| AT1G23935 | 3.672 | -0.231 | 0.029 |
| AT1G25320 | 4.546 | -0.101 | 0.011 |
| AT1G26240 | 3.699 | 0.161 | 0.028 |
| AT1G26560 | 3.553 | 0.120 | 0.033 |
| AT1G26810 | 4.798 | -0.119 | 0.008 |
| AT1G28520 | 3.712 | -0.053 | 0.028 |
| AT1G30825 | 5.122 | -0.127 | 0.006 |
| AT1G31020 | 3.551 | -0.072 | 0.033 |
| AT1G31330 | 3.540 | 0.383 | 0.033 |
| AT1G31420 | 4.620 | -0.084 | 0.010 |
| AT1G31750 | 4.998 | 0.150 | 0.007 |
| AT1G32361 | 3.884 | 0.045 | 0.023 |
| AT1G32580 | 3.966 | -0.087 | 0.021 |
| AT1G34120 | 3.785 | 0.121 | 0.025 |
| AT1G36100 | 3.571 | 0.055 | 0.033 |
| AT1G44910 | 3.286 | -0.076 | 0.043 |
| AT1G45180 | 4.439 | -0.186 | 0.012 |
| AT1G47317 | 3.303 | -0.072 | 0.043 |
| AT1G47603 | 4.229 | 0.157 | 0.016 |
| AT1G47640 | 3.423 | -0.074 | 0.038 |
| AT1G48690 | 4.456 | 0.190 | 0.012 |
| AT1G49130 | 3.404 | 0.052 | 0.039 |
| AT1G49900 | 5.520 | 0.144 | 0.003 |
| AT1G50240 | 3.305 | -0.154 | 0.042 |
| AT1G52565 | 5.544 | -0.106 | 0.003 |
| AT1G53750 | 3.810 | -0.102 | 0.025 |
| AT1G54290 | 3.776 | -0.071 | 0.025 |
| AT1G55090 | 3.685 | -0.126 | 0.028 |
| AT1G56150 | 3.547 | -0.219 | 0.033 |
| AT1G56150 | 4.127 | -0.123 | 0.018 |
| AT1G56260 | 3.269 | 0.102 | 0.044 |
| AT1G58190 | 6.307 | 0.130 | 0.002 |
| AT1G59650 | 3.219 | 0.136 | 0.047 |
| AT1G59790 | 4.870 | 0.064 | 0.008 |
| AT1G60460 | 3.433 | -0.088 | 0.037 |
| AT1G60710 | 3.743 | -0.120 | 0.027 |
| AT1G61270 | 3.881 | 0.178 | 0.023 |
| AT1G61700 | 3.405 | -0.027 | 0.039 |
| AT1G62380 | 3.463 | -0.217 | 0.036 |
| AT1G62440 | 5.276 | 0.282 | 0.005 |
| AT1G62500 | 3.175 | 0.151 | 0.049 |
| AT1G63060 | 3.992 | -0.144 | 0.020 |
| AT1G63360 | 3.453 | -0.040 | 0.036 |
| AT1G63450 | 3.537 | 0.117 | 0.033 |
| AT1G63900 | 4.012 | -0.052 | 0.020 |
| AT1G64380 | 3.173 | -0.073 | 0.049 |
| AT1G64600 | 5.174 | -0.105 | 0.006 |
| AT1G64620 | 5.987 | -0.087 | 0.003 |
| AT1G65190 | 3.449 | 0.211 | 0.036 |
| AT1G65450 | 3.373 | 0.061 | 0.040 |
| AT1G65570 | 4.050 | 0.248 | 0.019 |
| AT1G66200 | 4.399 | -0.248 | 0.013 |
| AT1G66750 | 3.316 | -0.078 | 0.042 |
| AT1G66970 | 3.837 | 0.141 | 0.024 |
| AT1G67090 | 4.294 | 0.345 | 0.015 |
| AT1G67455 | 3.849 | 0.074 | 0.024 |
| AT1G67600 | 3.517 | 0.056 | 0.034 |
| AT1G68210 | 3.161 | -0.080 | 0.050 |
| AT1G68250 | 3.813 | -0.089 | 0.025 |
| AT1G68300 | 4.152 | 0.163 | 0.017 |
| AT1G68910 | 3.673 | 0.100 | 0.029 |
| AT1G69820 | 4.687 | -0.056 | 0.009 |
| AT1G70260 | 3.222 | -0.303 | 0.046 |
| AT1G70490 | 3.462 | -0.178 | 0.036 |
| AT1G70520 | 3.652 | -0.175 | 0.029 |
| AT1G70810 | 3.193 | 0.098 | 0.048 |
| AT1G71130 | 3.357 | 0.090 | 0.040 |
| AT1G71250 | 3.384 | -0.131 | 0.039 |
| AT1G71697 | 5.110 | 0.117 | 0.006 |
| AT1G71800 | 5.052 | -0.045 | 0.006 |
| AT1G71900 | 4.154 | 0.047 | 0.017 |
| AT1G72210 | 3.319 | -0.146 | 0.042 |
| AT1G73160 | 3.827 | 0.115 | 0.024 |
| AT1G74390 | 3.458 | -0.155 | 0.036 |
| AT1G74650 | 5.667 | 0.070 | 0.003 |
| AT1G76680 | 3.165 | -0.238 | 0.049 |
| AT1G77170 | 4.200 | -0.104 | 0.016 |
| AT1G77720 | 4.764 | -0.138 | 0.008 |
| AT1G78210 | 3.838 | -0.090 | 0.024 |
| AT1G78310 | 3.970 | 0.208 | 0.021 |
| AT1G79420 | 3.758 | -0.072 | 0.026 |
| AT1G80680 | 3.798 | -0.174 | 0.025 |
| AT2G01080 | 4.489 | 0.064 | 0.011 |
| AT2G01505 | 3.690 | 0.156 | 0.028 |
| AT2G02620 | 5.680 | 0.124 | 0.003 |
| AT2G03210 | 5.538 | 0.106 | 0.003 |
| AT2G04060 | 4.534 | -0.092 | 0.011 |
| AT2G05210 | 4.163 | 0.065 | 0.017 |
| AT2G05210 | 4.533 | 0.065 | 0.011 |
| AT2G06050 | 3.946 | -0.149 | 0.021 |
| AT2G07827 | 3.197 | 0.057 | 0.048 |
| AT2G11851 | 4.930 | 0.189 | 0.007 |
| AT2G13660 | 4.142 | 0.265 | 0.017 |
| AT2G14170 | 3.396 | -0.148 | 0.039 |
| AT2G15430 | 3.273 | -0.177 | 0.044 |
| AT2G15620 | 3.178 | -0.231 | 0.049 |
| AT2G17180 | 4.292 | -0.067 | 0.015 |
| AT2G17190 | 4.040 | -0.130 | 0.019 |
| AT2G18100 | 5.774 | 0.069 | 0.003 |
| AT2G18180 | 3.208 | 0.114 | 0.047 |
| AT2G19470 | 4.153 | -0.074 | 0.017 |
| AT2G19500 | 3.412 | 0.098 | 0.039 |
| AT2G19520 | 3.889 | -0.103 | 0.023 |
| AT2G19650 | 3.557 | -0.169 | 0.033 |
| AT2G19700 | 3.556 | -0.032 | 0.033 |
| AT2G19820 | 3.795 | -0.041 | 0.025 |
| AT2G20520 | 3.197 | 0.209 | 0.048 |
| AT2G21560 | 3.174 | 0.200 | 0.049 |
| AT2G22610 | 3.250 | -0.181 | 0.045 |
| AT2G23600 | 3.533 | -0.152 | 0.033 |
| AT2G24400 | 3.321 | 0.080 | 0.042 |
| AT2G25730 | 3.696 | 0.034 | 0.028 |
| AT2G26800 | 4.508 | 0.103 | 0.011 |
| AT2G27420 | 4.411 | -0.070 | 0.013 |
| AT2G28210 | 3.619 | -0.155 | 0.030 |
| AT2G29470 | 3.283 | -0.311 | 0.043 |
| AT2G29620 | 3.601 | 0.154 | 0.031 |
| AT2G29670 | 3.669 | -0.290 | 0.029 |
| AT2G30660 | 6.420 | 0.123 | 0.002 |
| AT2G30970 | 3.191 | -0.261 | 0.048 |
| AT2G31320 | 3.513 | -0.102 | 0.034 |
| AT2G31370 | 3.256 | -0.122 | 0.045 |
| AT2G31670 | 5.265 | -0.195 | 0.005 |
| AT2G32530 | 3.605 | 0.110 | 0.031 |
| AT2G34357 | 3.372 | -0.107 | 0.040 |
| AT2G34660 | 3.336 | -0.127 | 0.041 |
| AT2G34870 | 3.903 | 0.089 | 0.023 |
| AT2G34930 | 3.998 | -0.182 | 0.020 |
| AT2G34970 | 3.167 | -0.106 | 0.049 |
| AT2G35000 | 3.414 | 0.190 | 0.038 |
| AT2G35200 | 4.173 | 0.069 | 0.017 |
| AT2G35480 | 3.337 | -0.167 | 0.041 |
| AT2G36380 | 3.467 | 0.206 | 0.036 |
| AT2G36895 | 3.887 | 0.125 | 0.023 |
| AT2G37160 | 4.953 | -0.075 | 0.007 |
| AT2G37920 | 3.929 | 0.092 | 0.022 |
| AT2G39690 | 3.652 | 0.124 | 0.029 |
| AT2G39970 | 4.419 | -0.148 | 0.013 |
| AT2G40030 | 3.295 | -0.213 | 0.043 |
| AT2G40080 | 3.220 | 0.120 | 0.047 |
| AT2G40200 | 3.508 | -0.103 | 0.034 |
| AT2G41350 | 3.534 | -0.161 | 0.033 |
| AT2G41390 | 3.929 | -0.107 | 0.022 |
| AT2G42680 | 4.135 | -0.059 | 0.018 |
| AT2G42840 | 3.884 | 0.201 | 0.023 |
| AT2G42870 | 3.178 | 0.167 | 0.049 |
| AT2G43460 | 3.782 | -0.093 | 0.025 |
| AT2G43760 | 3.197 | -0.122 | 0.048 |
| AT2G43990 | 3.977 | -0.070 | 0.020 |
| AT2G44160 | 3.890 | -0.127 | 0.023 |
| AT2G45060 | 3.250 | 0.053 | 0.045 |
| AT2G45500 | 3.361 | -0.052 | 0.040 |
| AT2G45850 | 3.686 | -0.039 | 0.028 |
| AT2G46550 | 3.972 | 0.232 | 0.021 |
| AT2G47240 | 3.352 | 0.111 | 0.040 |
| AT2G47360 | 3.383 | 0.183 | 0.039 |
| AT3G01140 | 4.262 | 0.083 | 0.015 |
| AT3G01290 | 3.530 | 0.075 | 0.033 |
| AT3G01970 | 3.557 | 0.092 | 0.033 |
| AT3G03170 | 4.663 | 0.240 | 0.009 |
| AT3G04980 | 3.769 | -0.189 | 0.026 |
| AT3G05155 | 3.678 | 0.125 | 0.029 |
| AT3G05180 | 4.218 | -0.070 | 0.016 |
| AT3G05400 | 5.392 | -0.163 | 0.004 |
| AT3G05550 | 3.261 | -0.165 | 0.045 |
| AT3G05710 | 3.246 | 0.066 | 0.045 |
| AT3G05980 | 4.179 | 0.089 | 0.017 |
| AT3G06040 | 5.549 | 0.089 | 0.003 |
| AT3G08490 | 3.290 | -0.101 | 0.043 |
| AT3G08840 | 3.285 | 0.092 | 0.043 |
| AT3G09090 | 3.647 | -0.092 | 0.029 |
| AT3G09770 | 4.064 | -0.142 | 0.019 |
| AT3G09780 | 4.442 | -0.120 | 0.012 |
| AT3G10605 | 5.592 | 0.089 | 0.003 |
| AT3G10850 | 3.263 | -0.085 | 0.044 |
| AT3G11110 | 3.234 | -0.090 | 0.046 |
| AT3G11470 | 4.262 | -0.088 | 0.015 |
| AT3G12780 | 3.497 | -0.235 | 0.035 |
| AT3G13090 | 3.335 | -0.202 | 0.041 |
| AT3G13225 | 3.374 | -0.040 | 0.040 |
| AT3G14330 | 3.786 | -0.088 | 0.025 |
| AT3G15355 | 3.616 | -0.039 | 0.030 |
| AT3G15720 | 3.329 | -0.230 | 0.042 |
| AT3G18630 | 3.711 | -0.125 | 0.028 |
| AT3G18710 | 3.507 | -0.108 | 0.034 |
| AT3G18730 | 3.308 | -0.215 | 0.042 |
| AT3G19180 | 3.388 | -0.097 | 0.039 |
| AT3G19800 | 6.330 | 0.032 | 0.002 |
| AT3G20100 | 3.895 | -0.063 | 0.023 |
| AT3G20340 | 3.762 | 0.242 | 0.026 |
| AT3G20520 | 4.495 | -0.445 | 0.011 |
| AT3G21090 | 4.498 | 0.071 | 0.011 |
| AT3G21550 | 3.421 | 0.137 | 0.038 |
| AT3G21710 | 5.323 | 0.164 | 0.004 |
| AT3G22425 | 3.894 | -0.199 | 0.023 |
| AT3G23190 | 3.620 | 0.112 | 0.030 |
| AT3G25560 | 3.234 | -0.139 | 0.046 |
| AT3G25620 | 3.316 | 0.153 | 0.042 |
| AT3G25950 | 3.902 | 0.161 | 0.023 |
| AT3G26740 | 3.311 | 0.286 | 0.042 |
| AT3G28410 | 3.376 | -0.051 | 0.040 |
| AT3G28580 | 3.264 | -0.224 | 0.044 |
| AT3G28670 | 4.214 | 0.064 | 0.016 |
| AT3G28910 | 4.654 | -0.144 | 0.009 |
| AT3G29250 | 3.356 | 0.155 | 0.040 |
| AT3G29631 | 3.891 | 0.100 | 0.023 |
| AT3G29636 | 4.266 | -0.080 | 0.015 |
| AT3G30823 | 3.445 | 0.072 | 0.037 |
| AT3G43540 | 3.589 | 0.057 | 0.032 |
| AT3G43572 | 3.591 | 0.059 | 0.032 |
| AT3G44235 | 5.380 | -0.062 | 0.004 |
| AT3G44670 | 3.644 | 0.067 | 0.029 |
| AT3G44720 | 3.253 | -0.111 | 0.045 |
| AT3G44890 | 3.167 | -0.070 | 0.049 |
| AT3G45240 | 4.058 | 0.088 | 0.019 |
| AT3G45980 | 3.852 | -0.123 | 0.024 |
| AT3G46450 | 3.387 | -0.105 | 0.039 |
| AT3G46790 | 4.032 | -0.124 | 0.020 |
| AT3G48150 | 4.323 | -0.092 | 0.014 |
| AT3G48640 | 3.539 | -0.140 | 0.033 |
| AT3G50120 | 3.163 | 0.136 | 0.050 |
| AT3G50430 | 4.168 | 0.114 | 0.017 |
| AT3G50530 | 4.894 | -0.069 | 0.008 |
| AT3G51360 | 3.900 | 0.154 | 0.023 |
| AT3G51600 | 3.290 | 0.122 | 0.043 |
| AT3G51960 | 4.015 | 0.236 | 0.020 |
| AT3G53320 | 4.045 | -0.158 | 0.019 |
| AT3G53400 | 3.183 | 0.102 | 0.049 |
| AT3G53520 | 4.211 | 0.081 | 0.016 |
| AT3G53900 | 3.713 | -0.082 | 0.028 |
| AT3G54630 | 3.309 | -0.086 | 0.042 |
| AT3G55720 | 3.429 | 0.142 | 0.038 |
| AT3G57080 | 3.299 | -0.030 | 0.043 |
| AT3G57340 | 4.777 | 0.117 | 0.008 |
| AT3G58560 | 3.713 | -0.110 | 0.028 |
| AT3G59620 | 5.727 | 0.076 | 0.003 |
| AT3G60350 | 3.295 | 0.044 | 0.043 |
| AT3G62030 | 3.251 | -0.287 | 0.045 |
| AT3G62180 | 3.739 | 0.047 | 0.027 |
| AT3G62570 | 4.240 | 0.113 | 0.016 |
| AT3G62580 | 3.579 | -0.073 | 0.032 |
| AT3G62770 | 3.992 | -0.114 | 0.020 |
| AT3G62960 | 3.158 | 0.040 | 0.050 |
| AT3G63220 | 3.520 | 0.115 | 0.034 |
| AT3G63490 | 4.392 | 0.128 | 0.013 |
| AT4G00026 | 4.846 | -0.060 | 0.008 |
| AT4G00170 | 3.534 | 0.120 | 0.033 |
| AT4G01450 | 3.321 | 0.071 | 0.042 |
| AT4G02075 | 5.092 | 0.181 | 0.006 |
| AT4G02640 | 7.098 | -0.060 | 0.001 |
| AT4G02800 | 3.248 | -0.193 | 0.045 |
| AT4G02870 | 4.121 | 0.105 | 0.018 |
| AT4G02980 | 3.663 | -0.104 | 0.029 |
| AT4G03090 | 4.048 | -0.108 | 0.019 |
| AT4G04900 | 4.187 | 0.096 | 0.017 |
| AT4G08395 | 4.725 | -0.067 | 0.009 |
| AT4G08790 | 5.644 | -0.115 | 0.003 |
| AT4G08874 | 3.997 | 0.051 | 0.020 |
| AT4G08950 | 4.430 | -0.468 | 0.012 |
| AT4G09170 | 4.860 | 0.063 | 0.008 |
| AT4G09630 | 3.305 | -0.070 | 0.042 |
| AT4G09647 | 3.659 | -0.054 | 0.029 |
| AT4G10290 | 4.017 | 0.068 | 0.020 |
| AT4G10320 | 4.071 | -0.166 | 0.019 |
| AT4G10340 | 4.504 | 0.234 | 0.011 |
| AT4G10620 | 4.850 | -0.119 | 0.008 |
| AT4G11150 | 3.194 | 0.132 | 0.048 |
| AT4G11760 | 3.467 | -0.077 | 0.036 |
| AT4G11790 | 3.334 | -0.101 | 0.042 |
| AT4G11900 | 3.721 | -0.054 | 0.027 |
| AT4G12140 | 5.777 | 0.028 | 0.003 |
| AT4G12790 | 5.726 | -0.115 | 0.003 |
| AT4G13830 | 3.559 | 0.152 | 0.033 |
| AT4G14070 | 3.255 | 0.094 | 0.045 |
| AT4G14104 | 5.462 | 0.179 | 0.004 |
| AT4G14145 | 3.679 | 0.117 | 0.029 |
| AT4G14190 | 4.322 | 0.059 | 0.014 |
| AT4G14880 | 3.395 | -0.084 | 0.039 |
| AT4G15233 | 6.183 | 0.128 | 0.002 |
| AT4G15440 | 3.788 | 0.044 | 0.025 |
| AT4G16260 | 4.928 | -0.199 | 0.007 |
| AT4G16740 | 3.364 | 0.073 | 0.040 |
| AT4G16850 | 4.733 | -0.132 | 0.009 |
| AT4G17470 | 4.849 | 0.329 | 0.008 |
| AT4G17490 | 4.301 | -1.968 | 0.015 |
| AT4G17490 | 3.684 | -0.807 | 0.028 |
| AT4G17640 | 4.734 | 0.081 | 0.009 |
| AT4G20150 | 3.972 | -0.106 | 0.021 |
| AT4G20410 | 3.309 | -0.054 | 0.042 |
| AT4G20930 | 3.491 | -0.169 | 0.035 |
| AT4G20990 | 4.168 | 0.115 | 0.017 |
| AT4G21070 | 3.682 | -0.199 | 0.028 |
| AT4G21860 | 3.730 | 0.120 | 0.027 |
| AT4G22120 | 5.056 | -0.175 | 0.006 |
| AT4G22160 | 4.506 | 0.092 | 0.011 |
| AT4G22580 | 3.232 | 0.112 | 0.046 |
| AT4G22770 | 3.464 | -0.220 | 0.036 |
| AT4G22990 | 3.537 | 0.108 | 0.033 |
| AT4G23580 | 3.869 | 0.056 | 0.023 |
| AT4G24175 | 4.066 | -0.174 | 0.019 |
| AT4G24190 | 3.430 | -0.129 | 0.038 |
| AT4G25100 | 5.656 | -0.244 | 0.003 |
| AT4G26100 | 5.379 | -0.214 | 0.004 |
| AT4G26650 | 3.464 | 0.130 | 0.036 |
| AT4G27420 | 3.667 | -0.074 | 0.029 |
| AT4G27440 | 3.209 | 0.210 | 0.047 |
| AT4G28490 | 4.118 | -0.064 | 0.018 |
| AT4G28780 | 4.834 | 0.017 | 0.008 |
| AT4G28811 | 3.605 | -0.242 | 0.031 |
| AT4G30210 | 3.551 | -0.166 | 0.033 |
| AT4G31600 | 4.022 | -0.114 | 0.020 |
| AT4G32290 | 3.744 | -0.079 | 0.027 |
| AT4G32460 | 4.723 | 0.145 | 0.009 |
| AT4G32540 | 3.202 | -0.133 | 0.048 |
| AT4G32680 | 4.350 | -0.076 | 0.014 |
| AT4G32830 | 3.210 | -0.223 | 0.047 |
| AT4G33670 | 5.343 | 0.119 | 0.004 |
| AT4G33800 | 3.226 | 0.139 | 0.046 |
| AT4G34040 | 3.526 | -0.121 | 0.033 |
| AT4G34500 | 5.049 | -0.061 | 0.006 |
| AT4G34800 | 3.181 | 0.224 | 0.049 |
| AT4G35780 | 3.827 | 0.126 | 0.024 |
| AT4G36220 | 3.312 | 0.174 | 0.042 |
| AT4G36970 | 3.473 | 0.174 | 0.036 |
| AT4G37000 | 4.128 | -0.096 | 0.018 |
| AT4G38120 | 3.169 | -0.064 | 0.049 |
| AT4G38160 | 3.367 | 0.145 | 0.040 |
| AT4G38210 | 3.462 | -0.200 | 0.036 |
| AT4G39380 | 3.370 | -0.072 | 0.040 |
| AT4G39690 | 3.986 | -0.064 | 0.020 |
| AT4G39980 | 3.282 | -0.198 | 0.043 |
| AT5G01560 | 3.271 | 0.157 | 0.044 |
| AT5G01890 | 3.321 | -0.123 | 0.042 |
| AT5G02160 | 3.546 | 0.171 | 0.033 |
| AT5G02330 | 4.021 | 0.106 | 0.020 |
| AT5G02850 | 3.556 | -0.067 | 0.033 |
| AT5G03720 | 3.424 | -0.145 | 0.038 |
| AT5G04347 | 3.194 | -0.084 | 0.048 |
| AT5G05365 | 4.014 | -0.232 | 0.020 |
| AT5G05670 | 3.335 | -0.154 | 0.041 |
| AT5G05740 | 3.264 | 0.192 | 0.044 |
| AT5G06360 | 4.854 | -0.079 | 0.008 |
| AT5G07260 | 4.512 | 0.051 | 0.011 |
| AT5G07390 | 3.807 | -0.189 | 0.025 |
| AT5G08410 | 4.275 | 0.079 | 0.015 |
| AT5G08505 | 4.558 | -0.058 | 0.011 |
| AT5G09300 | 3.876 | -0.157 | 0.023 |
| AT5G09330 | 3.847 | -0.047 | 0.024 |
| AT5G09995 | 3.282 | -0.171 | 0.043 |
| AT5G10120 | 4.020 | 0.121 | 0.020 |
| AT5G11060 | 3.480 | 0.051 | 0.036 |
| AT5G11160 | 5.262 | -0.247 | 0.005 |
| AT5G12330 | 3.405 | -0.031 | 0.039 |
| AT5G14450 | 3.492 | -0.234 | 0.035 |
| AT5G14930 | 3.312 | -0.127 | 0.042 |
| AT5G15000 | 4.377 | 0.059 | 0.013 |
| AT5G15510 | 3.430 | -0.161 | 0.038 |
| AT5G15700 | 4.995 | 0.074 | 0.007 |
| AT5G16110 | 3.281 | -0.199 | 0.043 |
| AT5G16490 | 4.192 | 0.054 | 0.017 |
| AT5G16740 | 3.406 | 0.052 | 0.039 |
| AT5G17960 | 4.882 | 0.296 | 0.008 |
| AT5G18040 | 3.409 | -0.153 | 0.039 |
| AT5G19570 | 3.180 | 0.113 | 0.049 |
| AT5G19730 | 3.249 | -0.162 | 0.045 |
| AT5G21150 | 3.542 | -0.094 | 0.033 |
| AT5G22910 | 3.911 | -0.042 | 0.022 |
| AT5G23010 | 3.569 | -0.265 | 0.033 |
| AT5G23110 | 4.453 | -0.041 | 0.012 |
| AT5G23470 | 3.258 | 0.037 | 0.045 |
| AT5G23580 | 3.191 | -0.148 | 0.048 |
| AT5G23680 | 3.168 | 0.062 | 0.049 |
| AT5G23750 | 4.845 | 0.172 | 0.008 |
| AT5G24010 | 3.446 | -0.196 | 0.037 |
| AT5G24600 | 3.173 | -0.287 | 0.049 |
| AT5G25820 | 3.457 | 0.103 | 0.036 |
| AT5G26110 | 3.849 | -0.150 | 0.024 |
| AT5G26751 | 3.209 | -0.151 | 0.047 |
| AT5G26805 | 3.570 | -0.069 | 0.033 |
| AT5G27610 | 3.624 | 0.131 | 0.030 |
| AT5G28310 | 3.450 | 0.083 | 0.036 |
| AT5G28442 | 4.089 | 0.127 | 0.019 |
| AT5G28590 | 3.563 | -0.107 | 0.033 |
| AT5G30520 | 3.170 | -0.033 | 0.049 |
| AT5G32613 | 3.319 | 0.105 | 0.042 |
| AT5G34830 | 3.521 | -0.026 | 0.034 |
| AT5G35580 | 3.606 | -0.204 | 0.031 |
| AT5G36160 | 4.656 | -0.167 | 0.009 |
| AT5G36870 | 3.802 | 0.060 | 0.025 |
| AT5G39630 | 3.200 | 0.100 | 0.048 |
| AT5G41080 | 3.199 | -0.075 | 0.048 |
| AT5G41890 | 3.239 | 0.090 | 0.045 |
| AT5G41940 | 3.724 | -0.106 | 0.027 |
| AT5G42580 | 3.293 | 0.214 | 0.043 |
| AT5G42740 | 3.362 | 0.102 | 0.040 |
| AT5G43420 | 3.386 | -0.155 | 0.039 |
| AT5G43755 | 3.330 | 0.076 | 0.042 |
| AT5G43810 | 4.312 | -0.079 | 0.014 |
| AT5G44005 | 3.182 | 0.263 | 0.049 |
| AT5G44610 | 3.257 | 0.287 | 0.045 |
| AT5G44620 | 4.733 | -0.060 | 0.009 |
| AT5G44670 | 4.061 | -0.163 | 0.019 |
| AT5G44740 | 5.775 | -0.123 | 0.003 |
| AT5G45780 | 3.391 | -0.155 | 0.039 |
| AT5G45800 | 4.049 | -0.150 | 0.019 |
| AT5G46310 | 3.180 | 0.054 | 0.049 |
| AT5G46450 | 3.277 | 0.105 | 0.044 |
| AT5G46590 | 6.469 | 0.114 | 0.002 |
| AT5G47175 | 3.930 | -0.081 | 0.022 |
| AT5G47450 | 4.502 | 0.247 | 0.011 |
| AT5G47480 | 3.627 | 0.152 | 0.030 |
| AT5G47970 | 3.735 | -0.069 | 0.027 |
| AT5G47990 | 3.161 | 0.368 | 0.050 |
| AT5G48570 | 3.469 | -0.191 | 0.036 |
| AT5G51450 | 4.370 | -0.043 | 0.013 |
| AT5G51520 | 3.397 | 0.158 | 0.039 |
| AT5G51970 | 3.260 | 0.155 | 0.045 |
| AT5G52070 | 3.284 | 0.175 | 0.043 |
| AT5G53710 | 4.730 | 0.145 | 0.009 |
| AT5G53800 | 3.648 | -0.150 | 0.029 |
| AT5G53830 | 4.868 | -0.219 | 0.008 |
| AT5G54070 | 3.939 | -0.039 | 0.021 |
| AT5G54850 | 6.491 | -0.132 | 0.002 |
| AT5G55130 | 3.676 | -0.076 | 0.029 |
| AT5G55170 | 3.189 | 0.144 | 0.048 |
| AT5G56300 | 3.550 | 0.083 | 0.033 |
| AT5G56760 | 3.578 | -0.126 | 0.032 |
| AT5G56760 | 5.580 | -0.114 | 0.003 |
| AT5G59020 | 4.079 | -0.131 | 0.019 |
| AT5G59800 | 3.488 | -0.101 | 0.035 |
| AT5G59840 | 3.283 | -0.091 | 0.043 |
| AT5G60170 | 4.837 | 0.087 | 0.008 |
| AT5G61250 | 3.288 | 0.111 | 0.043 |
| AT5G61680 | 3.590 | -0.099 | 0.032 |
| AT5G61960 | 3.542 | 0.055 | 0.033 |
| AT5G63030 | 5.392 | 0.211 | 0.004 |
| AT5G63860 | 4.091 | 0.125 | 0.019 |
| AT5G64930 | 3.846 | 0.167 | 0.024 |
| AT5G65080 | 6.970 | 0.213 | 0.001 |
| AT5G66010 | 4.664 | 0.069 | 0.009 |
| AT5G66530 | 4.588 | -0.097 | 0.010 |
| AT5G66710 | 4.313 | -0.125 | 0.014 |
| AT5G66920 | 5.328 | -0.097 | 0.004 |
| AT5G67270 | 3.672 | -0.242 | 0.029 |
